# A Powerful Paradigm for Cardiovascular Risk Stratification Using Multiclass, Multi-Label, and Ensemble-Based Machine Learning Paradigms: A Narrative Review

**DOI:** 10.3390/diagnostics12030722

**Published:** 2022-03-16

**Authors:** Jasjit S. Suri, Mrinalini Bhagawati, Sudip Paul, Athanasios D. Protogerou, Petros P. Sfikakis, George D. Kitas, Narendra N. Khanna, Zoltan Ruzsa, Aditya M. Sharma, Sanjay Saxena, Gavino Faa, John R. Laird, Amer M. Johri, Manudeep K. Kalra, Kosmas I. Paraskevas, Luca Saba

**Affiliations:** 1 Stroke Diagnostic and Monitoring Division, AtheroPoint™, Roseville, CA 95661, USA; 2 Department of Biomedical Engineering, North-Eastern Hill University, Shillong 793022, India; bhagawatimrinalini07@gmail.com (M.B.); sudip.paul.bhu@gmail.com (S.P.); 3 Research Unit Clinic, Laboratory of Pathophysiology, Department of Cardiovascular Prevention, National and Kapodistrian University of Athens, 11527 Athens, Greece; aprotog@med.uoa.gr; 4 Rheumatology Unit, National Kapodistrian University of Athens, 11527 Athens, Greece; psfikakis@med.uoa.gr; 5 Arthritis Research UK Centre for Epidemiology, Manchester University, Manchester 46962, UK; george.kitas@gmail.com; 6 Department of Cardiology, Indraprastha APOLLO Hospitals, New Delhi 110020, India; drnnkhanna@gmail.com; 7 Department of Internal Medicines, Invasive Cardiology Division, University of Szeged, 6720 Szeged, Hungary; zruzsa25@gmail.com; 8 Division of Cardiovascular Medicine, University of Virginia, Charlottesville, VA 22903, USA; as8ah@hscmail.mcc.virginia.edu; 9 Department of CSE, International Institute of Information Technology, Bhubaneswar 751003, India; sanjay@iiit-bh.ac.in; 10 Department of Pathology, A.O.U., di Cagliari-Polo di Monserrato s.s., 09045 Cagliari, Italy; gavinofaa@gmail.com; 11 Cardiology Department, St. Helena Hospital, St. Helena, CA 94574, USA; lairdjr@ah.org; 12 Department of Medicine, Division of Cardiology, Queen’s University, Kingston, ON K7L 3N6, Canada; amerjohri@gmail.com; 13 Department of Radiology, Massachusetts General Hospital, Boston, MA 02114, USA; mkalra@mgh.harvard.edu; 14 Department of Vascular Surgery, Central Clinic of Athens, N. Iraklio, 14122 Athens, Greece; paraskevask@hotmail.com; 15 Department of Radiology, A.O.U., di Cagliari-Polo di Monserrato s.s., 09045 Cagliari, Italy; lucasabamd@gmail.com

**Keywords:** CVD, multiclass, multi-label, ensemble, cloud, COVID, bias, gold standard

## Abstract

**Background and Motivation:** Cardiovascular disease (CVD) causes the highest mortality globally. With escalating healthcare costs, early non-invasive CVD risk assessment is vital. Conventional methods have shown poor performance compared to more recent and fast-evolving Artificial Intelligence (AI) methods. The proposed study reviews the three most recent paradigms for CVD risk assessment, namely multiclass, multi-label, and ensemble-based methods in (i) office-based and (ii) stress-test laboratories. **Methods:** A total of 265 CVD-based studies were selected using the preferred reporting items for systematic reviews and meta-analyses (PRISMA) model. Due to its popularity and recent development, the study analyzed the above three paradigms using machine learning (ML) frameworks. We review comprehensively these three methods using attributes, such as architecture, applications, pro-and-cons, scientific validation, clinical evaluation, and AI risk-of-bias (RoB) in the CVD framework. These ML techniques were then extended under mobile and cloud-based infrastructure. **Findings:** Most popular biomarkers used were office-based, laboratory-based, image-based phenotypes, and medication usage. Surrogate carotid scanning for coronary artery risk prediction had shown promising results. Ground truth (GT) selection for AI-based training along with scientific and clinical validation is very important for CVD stratification to avoid RoB. It was observed that the most popular classification paradigm is multiclass followed by the ensemble, and multi-label. The use of deep learning techniques in CVD risk stratification is in a very early stage of development. Mobile and cloud-based AI technologies are more likely to be the future. **Conclusions:** AI-based methods for CVD risk assessment are most promising and successful. Choice of GT is most vital in AI-based models to prevent the RoB. The amalgamation of image-based strategies with conventional risk factors provides the highest stability when using the three CVD paradigms in non-cloud and cloud-based frameworks.

## 1. Introduction

Cardiovascular disease (CVD) results in 18 million deaths worldwide [1]. In 2020, the financial burden due to CVD was $237 billion USD [2]. With COVID-19 still not subsided, rising inflation costs, loss of families due to migration, depression on the rise, and comorbidities increasing, the risk of CVD risk is likely to go up. The main cause of CVD is atherosclerotic deposition in the heart’s coronary arteries [3]. Due to different types of comorbidities such as diabetes [4], chronic kidney disease (CKD) [5,6], rheumatoid arthritis [7,8], hypertension [9], high lipids [10], and brain diseases [11,12,13], the risk of CVD is increasing, putting patients at a higher risk of heart disease and stroke. It is estimated that by 2030, the financial burden due to CVD will reach about $3T USD [2]. Therefore, the need for an early CVD risk detection system will alleviate the mortality and morbidity rates.

CVD risk assessment can take two forms, namely (a) in the doctor’s office or pathology laboratory or both, (b) in the stress test centers or signal processing clinics [14,15,16]. The calculators used in the office-based scenario are conventional CVD calculators that use laboratory-based biomarkers (LBBM) and office-based biomarkers (OBBM) [17], while the CVD risk assessment in stress test centers uses electrocardiograms (ECG) [18,19,20]. There are multiple conventional tools for assessment of risk due to CVD, namely (i) QRISK3 [21], (ii) Framingham risk score (FRS) [22], (iii) the systematic coronary risk evaluation score (SCORE) [23], (iv) the Reynolds risk score (RRS) [24], and (v) the atherosclerosis cardiovascular disease (ASCVD) [25]. Specific guidelines like the American College of Cardiology/American Heart Association (ACC/AHA) [26], the European Society of Cardiology (ESC) [27,28], and the Canadian society [29,30] are followed for predicting the CVD risk when using these calculators.

The conventional CVD calculators offer several challenges [26,27], which include (i) not being able to deal with the non-linearity between the covariates (or risk factors) [31] and the gold standard (outcomes); (ii) does not reflect a direct representation of plaque build-up in the arteries [17,32,33]; (iii) usage of ad hoc threshold for CVD risk stratification and lack granularity for CVD [34,35]; and (iv) finally, the lack of usage of cohort’s knowledge. All the abovementioned reasons put pressure to investigate a more accurate CVD risk classification tool that can assess the proper non-invasive atherosclerotic plaque burdens by using LBBM and OBBM.

**Figure A8:**
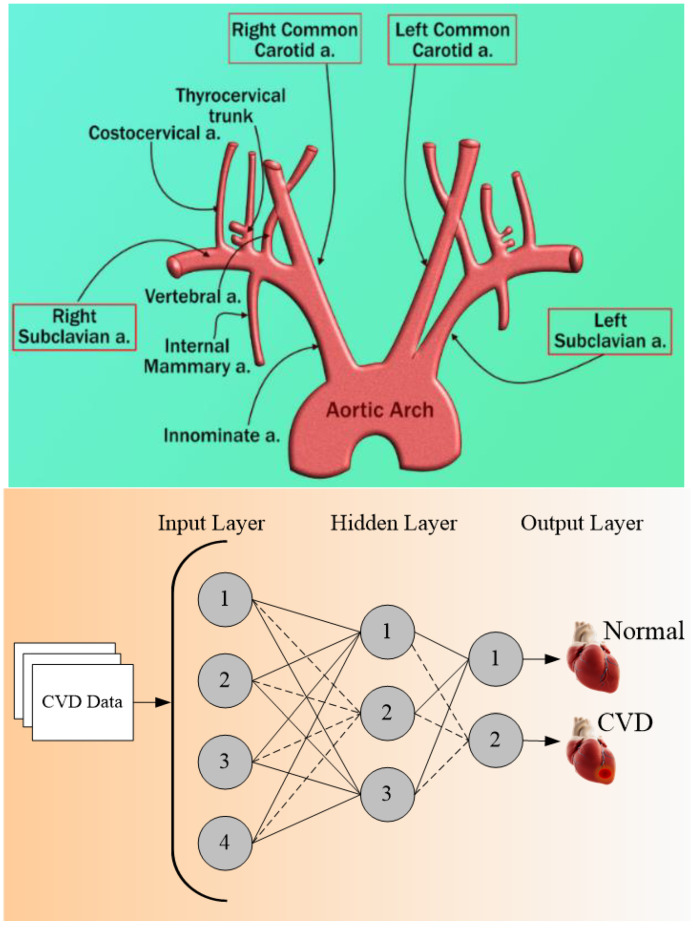
(**Top**) Anatomical link between the carotid artery and aortic arch. (**Bottom**) Typical neural network for CVD risk stratification.

When it comes to a non-invasive framework, the risk of coronary artery disease can be estimated via the carotid artery network, because of the same genetic composition of these two arteries (see Appendix H, Figure A8: Top). Carotid artery imaging also provides an advantage to both CVD and stroke risk predictions and is often adapted to act as a surrogate type of biomarker for CVD risk classification [36]. Generally, for imaging, the carotid arteries, the popular three medical imaging modalities used are magnetic resonance imaging (MRI) [37], computed tomography (CT) [38], and ultrasound (US) [39]. 

Carotid B-mode ultrasound (cBUS) offers several benefits, namely cost-effectiveness, user-friendliness, easy reach through the neck window, high-resolution via compound, and harmonic imaging [39,40,41]. Carotid videos can be also generated in the form of movies (so-called CINIE loop with cardiac gating) during imaging, which can then be used for better carotid plaque vulnerability. This can be accomplished by correlations and characterization [42] by taking the advantage of image registration paradigms between the slices. The phenotypes for carotid ultrasound image-based (CUSIP) technique are carotid intima-media thickness (cIMT) [43,44,45,46,47], intima-media thickness variability (IMVT) [48,49,50,51], maximum plaque height (MPH) [52,53,54], and total plaque area (TPA) [55,56,57] and can be obtained using cBUS frozen scans. The classification of risk for CVD can be improved in terms of more reliable results by fusing CUSIP biomarkers along with the OBBM, LBBM as shown by AtheroEdge 2.0 (Roseville, CA, USA) [36]. Though it is fully automated and statistically based, it does not use cohort’s knowledge and Artificial Intelligence (AI) framework. Therefore, a more accurate solution is needed to handle this challenge to ensure reliable and superior CVD risk prediction.

With the advancement of AI in the field of healthcare [19,58,59,60,61,62], especially in machine learning (ML), deep learning (DL), combined with mobile solutions such as e-health and cloud-based technologies, CVD risk assessment has shown promising signs. The main focus of the proposed study is the ML paradigm however, we very briefly touch on DL strategies due to their infancy stage. Recently, we have seen research showing that ML can handle non-linearity between the input covariates and target outcomes (or gold standard), while DL automates the feature extraction process from the input data for classification. We therefore hypothesize that CVD classification paradigms such as multiclass, multi-label, and ensemble are more accurate and reliable. Due to the amalgamation of the linear and non-linear covariates along with the gold standard, there is no clear-cut defined strategy when adapting these three paradigms for CVD risk stratification. This can sometimes lead to over-performance inaccuracies and under-performance in clinical outcomes leading to bias in AI [63]. The proposed study also presents the bias measurements in these three paradigms independently, and further when all the three sets of techniques are jointly taken into consideration for CVD risk stratification. The pseudo-code for each technique is discussed in Appendix A, Appendix B and Appendix C. With the evolution of fast-growing telecommunication technology, these CVD techniques can be applied in e-health frameworks such as mobile or cloud settings, which provide access to the patient population for rural areas of the world. This review further dwells in the above-mentioned area. Lastly, due to changing environmental conditions such as COVID-19, it is important to understand how the CVD risk assessment integrates into the COVID-19 framework. Several CVD reviews are already available [64,65,66,67,68,69], but none of these consider the recent advanced methods like using ML and DL in office-based, mobile/cloud-based set-ups.

The design of the proposed review is as follows. Section 2 shows the PRISMA strategy used for study selection along with the statistical distribution of AI attributes. Section 3 presents the biological link between atherosclerosis and CVD risk. Section 4 represents the heart of the system discussing the three paradigms, namely multiclass, multi-label, and ensemble-based CVD risk stratification along with performance evaluation (PE) metrics for these techniques. Section 5 presents the bias in AI for these three methods. The CVD risk assessment through mobile, e-Health, and cloud-based techniques is presented in Section 6. The critical discussion of the review is in Section 7, while the study concludes in Section 8.

## 2. Search Strategy and Statistical Distributions

The statistical distribution of the literature is necessary to understand the types of CVD methods, the gold standard adapted for these AI-based solutions, the participation of the feature extraction methods, and bias in the AI-based solutions. Thus, we adapt the PRISMA model for the selection of the studies for the CVD risk assessment. This section is therefore divided into two parts: Section 2.1 discusses the study selection criteria and Section 2.2 presents the statistical distributions.

### 2.1. PRISMA Model

**Figure 1:**
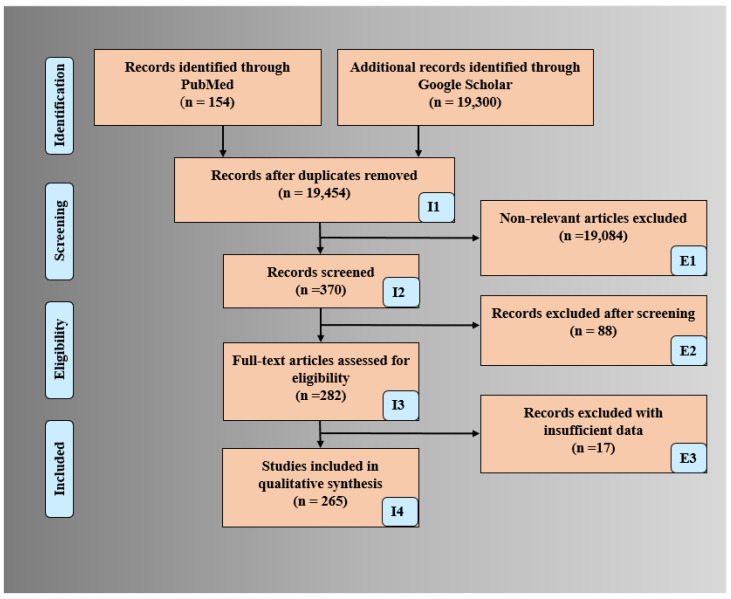
PRISMA model for selection of studies for CVD risk assessment.

The PRISMA model was used for searching and selecting the final studies for the review. The search was done using Science Direct, Google Scholar, IEEE Xplore, and PubMed by adapting the following keywords “multiclass classification for CVD risk”, “multi-label classification for CVD risk”, “ensemble classification for CVD risk”, “CVD risk using Machine Learning/Artificial Intelligence for multiclass”, CVD risk using Machine Learning/Artificial Intelligence for multi-label, “CVD risk using Machine Learning/Artificial Intelligence for ensemble”, “CVD risk assessment in ML/AI framework”, and “Bias in ML/AI”. The total number of ML/AI-based CVD studies is shown in Figure 1. An exhaustive search resulted in a total of 19,454 studies. The three criteria used for exclusion were (a) non-relevant studies (b) articles removed after search and screening of the studies (c) records rejected due to insufficient data. The implementation of exclusion criteria provides 19,084, 88, and 17 studies for exclusion showed by E1, E2, and E3 (Figure 1). The important scientific knowledge from these final studies was gained and the statistical classification was drawn. Further, a comprehensive analysis of the studies was done between the three techniques with the determination of AI bias.

### 2.2. Statistical Distribution

**Figure 2:**
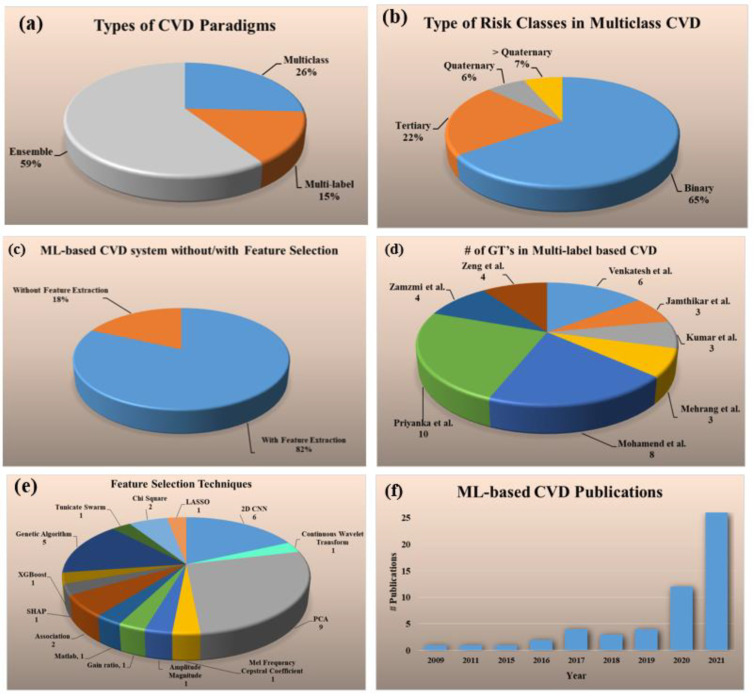
Statistical distribution (**a**) types of CVD paradigms, (**b**) types of risk classes in multiclass CVD (**c**) ML-based CVD systems without/with feature selection, (**d**) # GT’s in multi-label based CVD, (**e**) feature selection techniques, (**f**) trend of the ML-based CVD publications by year.

The statistical distributions derived from the selected studies are shown in Figure 2. The following attributes were used for the statistical distribution (a) types of CVD paradigms, (b) types of risk classes in multiclass CVD (c) ML-based CVD systems without/with feature extraction, (d) # GTs in multi-label-based CVD, (e) feature selection techniques, and (f) ML-based CVD publications.

The percentage of studies for each of the three kinds of CVD risk prediction had the following distributions: multiclass (26%) [69,70,71,72,73,74,75,76,77,78,79,80,81,82], multi-label (15%) [83,84,85,86,87,88,89,90], and ensemble (59%) [80,91,92,93,94,95,96,97,98,99,100,101,102,103,104,105,106,107,108,109,110,111,112,113,114,115,116,117,118,119,120,121] (Figure 2a). Several different kinds of risk classes were identified in multiclass CVD framework, namely binary (65%), tertiary (22%), quaternary (6%), and greater than quaternary (7%) (Figure 2b). The distribution of the ML-based CVD studies with and without feature selection are shown in Figure 2c. It was found that almost 82% of ML-based CVD studies performed feature selection for risk prediction whereas only 18% [69,70,73,75,83,94,96,110,120] did not perform it. For the ML-based multi-label CVD (Figure 2d), the total number of GT’s used for each study were as follows and given in the ground braces: Venkatesh et al. (6) [83], Jamthikar et al. (3) [84], Kumar et al. (3) [85], Mehrang et al. (3) [86], Mohamend et al. (8) [87], Priyanka et al. (10) [88], Zamzmi et al. (4) [89], and Zeng et al. (4) [90]. There were eight sectors in the pie chart and each sector represents a study (publication) in the area of multi-label-based ML system. Below the study shows the number of gold standards used for the design of the multi-label ML system paradigm. For example, Ventakesh et al. had 6 types of gold standards ((death, stroke, coronary heart disease (CHD), CVD, heart failure (HF), atrial fibrillation (AF)) during the design of their multi-label-based ML system. Similarly, Jamthikar et al. had three types of gold standard (coronary artery disease (CAD), acute coronary syndrome (ACS), composite cardiovascular event (CVE)) during the design of the multi-label ML system. Since the number of gold standards are important during the multi-label paradigm, the pie-chart shows the statistical distribution of the different studies using the number of gold standards. The number of studies (given in curly braces) that used the following feature selection techniques were 2D convolutional neural network (CNN) (6) [71,79,81,89,101,111], continuous wavelet transform (1) [72], principal component analysis (PCA) (9) [76,79,84,98,102,112,114,119,121], Mel frequency cepstral coefficient (1) [77], amplitude magnitude (1) [78], gain ratio (1) [80], Matlab (1) [86], association technique (2) [87], SHAP (1) [90], extreme gradient boost (XGBoost) (1), genetic algorithm (5) [91,103,104,122,123], Tunicate Swarm (1) [116], chi-Square (2) [117], least absolute shrinkage and selection operation (LASSO) (1) [99] (Figure 2e). The increasing trend of CVD publications from the year 2009 to 2021 is shown in Figure 2f.

## 3. Biological Link between Atherosclerosis and Cardiovascular Disease

**Figure 3:**
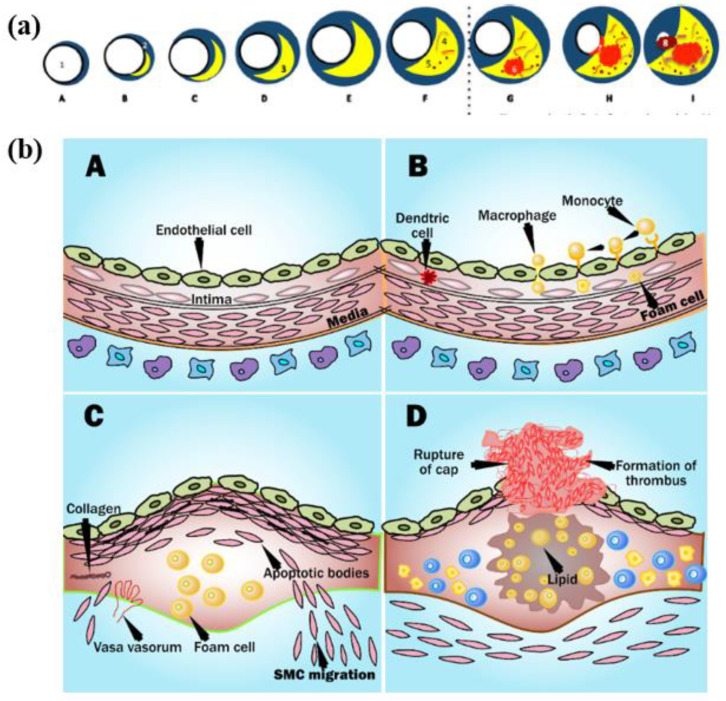
(**a**) Plaque formation in the coronary artery and (**b**) process of plaque rupture in coronary artery (Courtesy of AtheroPoint™, Roseville, CA, USA) [131].

The fundamental cause of CVD is the disease of atherosclerosis [124]. The process of plaque formation is known as atherogenesis as shown in Figure 3a(A–I) [125]. It is a process when the plaques develop in the arteries where there is low endothelial shear stress [126]. The shear stress depends on the flow velocity characteristics like type of flow, direction, and velocity. Leukocytes attack the epithelium in this region (Figure 3(bA)) [126]. Mainly there is the migration of monocytes into the sub-epithelial layer where it is oxidized by the low amount of low-density lipoprotein (LDL) cholesterol and turns into macr0ophage (Figure 3(bB)) [127,128]. Eventually, these macrophages become large foam cells with oxidized LDL cholesterol leading to the formation of necrotic core (Figure 3(bC)). Microscopic calcium granules expand in the necrotic cells and forms lumps of calcium deposits. This necrotic core is separated from the blood vessel by a fibrous cap [129]. The blood remains uninterrupted when the plaque is small as the arteries do remodeling by themselves [130]. However, when the plaques increase, the lipid-core volume decreases leading to structural stabilization of plaque (Figure 3a) [131].

Progressive deposition of lipids results in the thinning of the fibrous cap leading to rupturing the plaque [132]. The ruptures of the cup result in healing by the platelets in the bloodstream, which leads to the formation of the clot of blood or thrombus which yields blocking of artery than atrial stiffness [133]. Due to this, the tissues become deprived of blood supply, causing cell death. If the coronary artery gets blocked, causing a myocardial infarction or CVD (Figure 3(bD)) [3,7].

## 4. Three Paradigms for Cardiovascular Risk Stratification

The core aim of this review is to understand the three kinds of paradigms for CVD risk stratification. This allows understanding the (a) types of gold standards used for different kinds of applications, (b) types of fundamental architectures used, and (c) finally the comparison between the three different types of paradigms.

### 4.1. Multiclass-Based Cardiovascular Disease Risk Stratification System

The most fundamental type of CVD risk stratification is the multiclass framework [134]. There are three main characteristics in multiclass framework, namely (i) it divides the outcome into two or more granular risk classes, (ii) the drug prescription is better controlled for CVD treatments based on which class the disease stage or risk lies, and (iii) the risk of CVD is better understood when divided into several stages such as low, mild, low-of-moderate, high-of-moderate, low-of-high, and high-of-high.

#### 4.1.1. CVD-Based Multiclass Risk Assessment System

For any CVD system, there are two most important attributes: (a) the types of the covariates used and (b) the gold standard adopted. Accordingly, in the multiclass framework, there are 14 published studies (see Table 1). It shows the three attributes represented in three columns: covariates, gold standard, and the AI category, namely ML or DL. The types of covariates considered for the multiclass systems were OBBM [71,76,80,82], LBBM [71,76,80,82], CUSIP [76,80] for *office-based setups* (Table 1: row 1–5), and Electrocardiogram (ECG) [79,81,82], PCG [77], Acceleration Plethysmogram (APG) [78] signals for *cardiac stress test laboratories* (Table 1: row 6–9), and coronary artery calcium (CAC) for CT-based CVD models [135]. The ground truths considered for CVD risk assessment (Table 1) were death [80], coronary heart disease (CHD) [82], chronic heart conditions (CHC) [79], cardiovascular event (CVE) [76], sudden cardiac death (SCD) [72], heart failure (HF), myocardial infarction (MI) [75], coronary artery calcification (CAC) score [69], fatal/non-fatal CVD [73], joint CVD and diabetes [70]. Note that these gold standard choices along with AI attributes, scientific and clinical validations are key to preventing bias in AI.

#### 4.1.2. Comparison between CVD Application and Non-CVD Application

The comparison between CVD and non-CVD applications [136] is shown in Table 2. Seven attributes were used for this comparison. The image modalities used in the CVD-based system were US, CT, MRI, and ECG (Table 2: row 4, CVD column). The architectures applied were ML and DL. DL provided better results due to its unique automated feature selection process (Table 2: row 6, CVD column). The defined number of classes was in the range of 3–9 (Table 2: row 5, CVD column) [69,70,71,72,73,74,75,76,77,78,79,80,81,82]. The multiclass approach for classification has been applied to non-CVD applications such as Alzheimer’s prediction or different cancer types. The interpretation of multiclass in the non-CVD system can be thought of as different stages of the diseases, for example, in the case of Alzheimer’s disease (AD), it can be categorized as different stages of memory loss with age. Similarly, in the case of cancer, it can be different stages or grades of cancer.

Our observations show that the gold standard types in the non-CVD system are very different from the CVD systems. For example, for the early detection of AD/Mild Cognitive Impairment (MCI), the classification is done between (1) AD vs. normal control (NC), (2) MCI vs. NC, (3) AD vs. MCI, and (4) progressive MCI (PMCI) vs. Significant Memory Concern (SMCI) for Alzheimer’s. In the case of breast cancer, GTs can be proliferation and non-proliferation cancer types. 

Note that the number of classes considered for multiclass differs from disease-to-disease. The different architecture followed for CVD are mainly ML and DL, whereas for non-CVD it ranges from deep learning retinal CAC score (RetiCAC) [137], pooled cohort equation (PCE) [138,139], support vector machine (SVM) [70,75,76,77,140], convolutional neural networks (CNN), decision tree (DT) [71,79], random forest (RF), logistic regression (LR), naive Bayesian (NB), K-nearest neighbor (KNN), and ensemble. The different types of covariates for no-CVD-based systems were breast histopathology images (BHI), OBBM, and LBBM (Table 2: row 2, column non-CVD). Modalities for the non-CVD-based system were EEG, MRI, CT [137,139] (Table 2: row 4, non-CVD column), and the number of risk classes varied from 5–14 [137,138,139,141,142] (Table 2: row 5, non-CVD column).

#### 4.1.3. Multiclass CVD Architecture for Office-Based CVD Risk Stratification

The architectures opted for multiclass prediction of CVD risk has very basic components (a) data collection (b) training system, and (c) testing system. The training system is basically used for training the ML system based on different covariates (or risk factors) [143,144], with the support of different ground truths while using the training-based classifiers. The system can be trained to identify the granular risk classes from no, low, and medium, to high class. Feature selection is also performed during the training of the system [145,146]. For prediction, the training model is applied to transform the testing features either in Seen AI framework or the Unseen AI framework [147]. Two types of architectures were described in this section in terms of the above-mentioned factors. A typical online system for multiclass CVD risk stratification is shown in Appendix A, Appendix A.1.

**Figure 4:**
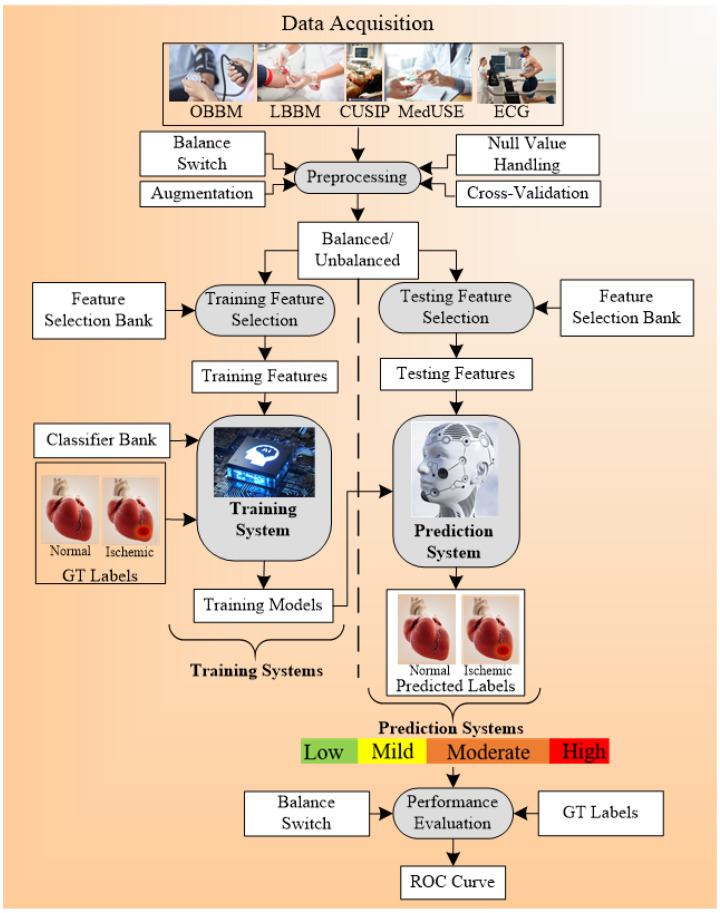
Multiclass architecture for CVD risk stratification (AtheroEdge 3.0_ML_).

A generalized ML system is applied to *office-based* CVD or stress-test-based CVD systems as shown in Figure 4. Considering the *office-based* CVD system, the covariates were collected from OBBM, LBBM, CUSIP, and MedUSE [76], while for the CVD-based stress-test system, EEG was the input. The rest of the configuration remains the same which consists of four parts: Part A is the preprocessing of the input data (covariates) and augmentation for balancing the classes. Part B consists of a training system, Part C consists of a prediction system, and Part D consists of a performance evaluation system (Appendix E). In Part A, the objective is to balance the classes if there is a multiclass scenario, Part B consists of two subparts: (i) selection of the best feature given the set of covariates and (ii) model generation using (a) classifier, (b) selected features, and the (c) gold standard. Part C consists of the application of the trained model on the selected set of best features from the test data set by transforming the test features to compute the predicted label. Part D is used for performance evaluation of the ML system where the predicted labels are compared against the gold standard labels. Note that during the training system, the two ingredients are the classifier bank and the gold standard used. The classifier bank, for example, can be classifiers like SVM, XGBoost, KNN, NB, etc., while the gold standard is the coronary artery disease syndrome, such as coronary artery disease stages that include the four types of risk stages. Note that since the system is a K-fold (either of the K types such as K2, K3, K4, K5, and K10 can be used), every patient gets to be in the test pool, and then at the end of all the folds, the complete set can be used for performance evaluation. Further to note a classifier bank can be used during the design of the training model, that uses the gold standard (such as coronary risk scores derived from coronary angiography) and training covariates. The CVD example in Figure 4 uses four sets of covariates, which can be flipped to ECG signals [148,149,150] when using the stress test-based system for CVD risk assessment. The longitudinal ultrasound model is used typically for the collection of the CUSIP risk factors such as cIMT (max., min., and ave.), intima-media thickness variability (cIMTV), maximum plaque height (MPH), and total plaque area (TPA). 

#### 4.1.4. Multiclass CVD Architecture for Cardiac Stress Laboratories

**Figure 5:**
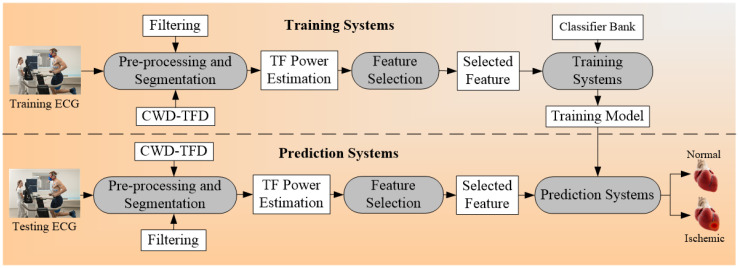
Example of multiclass architecture; CWD: Choi-William’s time-frequency distribution; TF: time-frequency.

Another set of architecture for multiclass CVD risk prediction was used by Hussein et al. [75] (Figure 5). The ECG signals [151,152,153] are obtained from the stress test laboratory for the analysis of CVD risk. The model uses the multiclass SVM classifier that takes the ECG signals as risk factors or covariates. And the ground truth used for the training system is myocardial infarction (MI). The multiclass outcomes that were identified were normal, low MI, and high MI. The feature of ST (it is the interval between ventricular depolarization and repolarization, and PR (the flat line that runs from the end of the P-wave till the start of the QRS complex) were extracted from the time-frequency (TF) power spectrum. The created training model was the input to the prediction systems along with the test data and the final classifications were made into the normal, low MI, and high MI.

The general algorithm for multiclass CVD risk stratification is explained in form of pseudo-code. A detailed explanation is provided in Appendix A, Appendix A.2.

### 4.2. Multi-Label-Based Cardiovascular Disease Classification

The second technique used for CVD risk stratification is multi-label-based [154,155,156]. The ground truth is very important for the proper classification of CVD risk [157,158,159]. CVD risk prediction systems were said to be multi-label-based depending on the number of ground truth (GT) used in the system [160,161,162]. The paradigm was considered as a multi-label-based classification if more than one number of GT is used for CVD risk detection [90,163,164,165,166,167]. The GTs, risk factors, and the architecture used were discussed in the next sub-sections. The pseudo-code that represents a multi-label-based risk stratification process can be referred to in Appendix B.

#### 4.2.1. Covariates and Risk Factors for Multi-Label-Based CVD Classification

Eight multi-label-based studies for CVD risk prediction were considered in this review [83,84,85,86,87,88,89,90]. Different types of ground truths used in these studies were death, stroke, CHD, CVD, HF, atrial fibrillation (AF) [83], CAD, ACS, composite CVE [84], large vessel disease (LVD), small vessel disease (SVD) [168], intracerebral hemorrhage (ICH) [85], non-AFib-non-ADHF, AFib-non-ADHF, AFib-ADHF [86], systolic heart failure (acute, chronic type), diastolic heart failure (acute and chronic type) [87], congestive heart failure, hypertension, AF, acute kidney failure, diabetes type II, acute respiratory failure, hyperlipidemia, coronary atherosclerosis, urinary tract infection, esophageal reflux [88], CAD, dilated cardiomyopathy (DCM), MI [89], lung complication, cardiac, infectious and rhythmic complication [90]. 

The risk factors used were OBBM, LBBM, CUSIP, MRI, and CT image phenotypes (input covariates column, Table 3). The algorithms used for the multi-label classifications were namely binary recursive (BR), label powerset (LP), multi-label adaptive resonance associative map (MLARAM), random k-labelset (RakEL), classifier chain (CC), multi-label k-nearest neighbor (MLkNN), seismocardiography (SCG-Z), gyrocardiography (GCG-Z), principal component analysis (PCA), DCT, consensus-based risk model. Other characteristics of this classification technique were described in Table 3.

#### 4.2.2. Multi-Label-Based Architectures for CVD Risk Stratification

**Figure 6:**
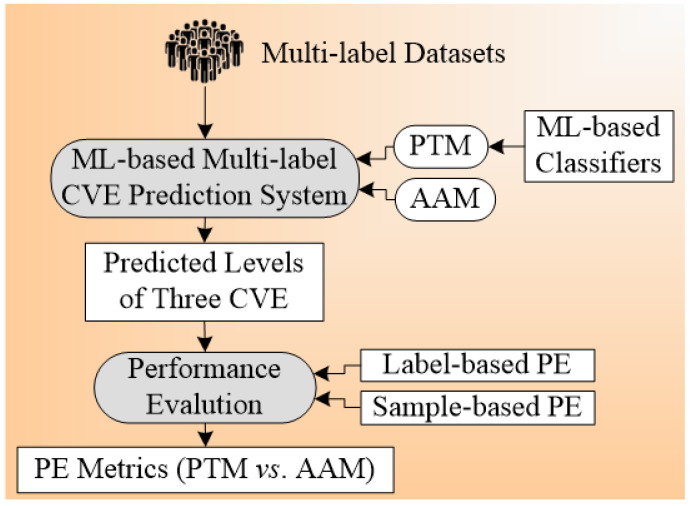
Architecture for multi-label-based CVD risk classification using carotid ultrasound.

The architecture design for the multi-label plays an important in the outcome results of the system. The basic component of the architecture for the CVD prediction system is training and testing. The proper choice of GT leads to non-biased results in the risk prediction of CVD. The architecture system used by Jamthikar et al. [84] is shown in Figure 6 below. The total number of ground truths considered for this system were three, namely (a) coronary artery disease, (b) acute coronary syndrome, and (c) a composite CVE, and the covariates used were OBBM, LBBM, and the CUSIP phenotype. Six types of classification techniques used include (i) four problem transformation methods (PTM) and (ii) two algorithm adaptation methods (AAM) are used for multi-label CVE prediction. The four PTM techniques were binary relevance (BR), label powerset (LP), classifier chain (CC), and random k-labelset (RAkEL). Under AAM-based, two techniques, namely multi-label k-nearest neighbor (MLkNN), and multi-label adaptive resonance associative map (MLARAM) were used. The details can be seen in Appendix B. Evaluation was performed by calculating the accuracy, sensitivity, specificity, F1-score, and AUC for all the classification techniques. The BR classification was found to be the best performer with the values for accuracy, sensitivity, specificity, F1-score, and AUC as 81.2%, 76.5%, 83.8%, 75.37, and 0.89 (*p* < 0.0001), respectively.

**Figure 7:**
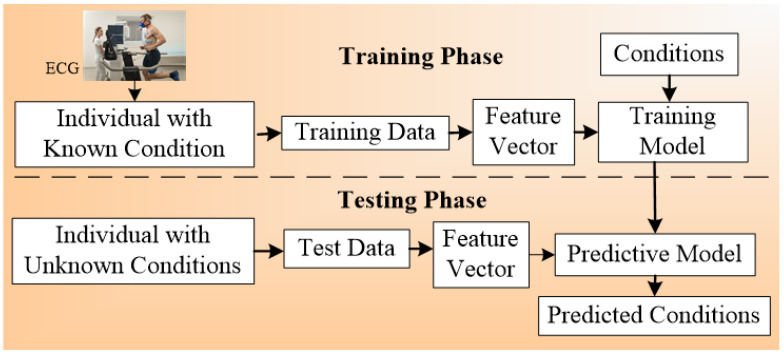
ECG architecture for multi-label-based CVD classification.

Another architecture [86] used for multi-label CVD classification is described in Figure 7. The mechanocardiography (MCG) data were used by the system. Four kinds of ground truth were used, namely AFib, non-AFib, ADHF, and non-ADHF. The covariates were gender, age, height, weight, BMI, given for the training and testing system. The ML classification algorithm used were random forest (RF), Xtreem Gradient Boost (XGB), and logistic regression (LR). RF gave the best performance among all the three ML classifiers. The system was validated by nested cross-validation. In this system, feature extraction was also performed using a feature vector. The hierarchal classification was also adapted in this system. Another paradigm that can use multiple classifiers at the same time is under the ensemble framework as presented in the next section.

### 4.3. Ensemble-Based Cardiovascular Disease Classification

**Figure 8:**
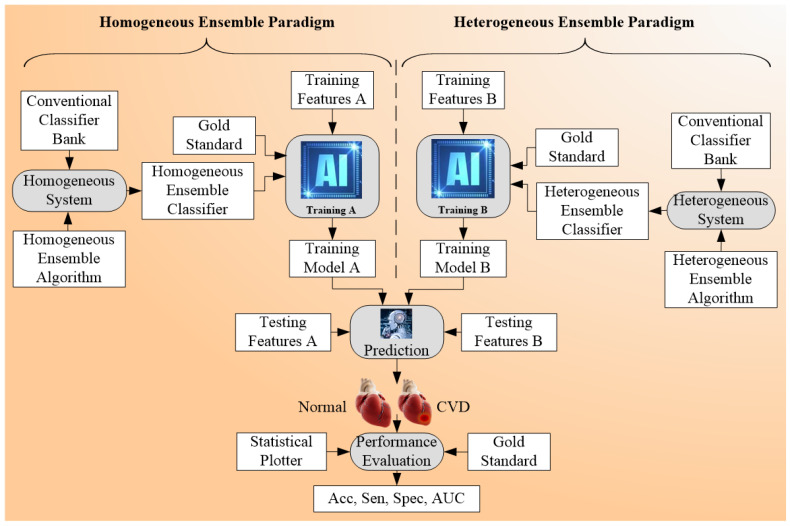
Ensemble-based Architecture for CVD risk stratification.

The ensemble-based technique was the third type of technique considered for CVD risk classification [169,170,171]. This classification was characterized by the fusion of different types of ML or DL classifiers (Table 4). It can be used with multiclass and multi-label classification [172,173,174]. Figure 8 shows the concept of the ensemble paradigm. There are two sets of strategies, namely homogeneous ensemble and heterogeneous ensemble (see the separation shown by dotted line). In homogenous ensemble, the conventional classifier techniques are combined using homogeneous ensemble algorithm to yield homogeneous ensemble classifier, which when trained using classifier A while using the gold standard. This homogeneous system yields the trained model A. The same protocol can be adapted for the heterogeneous ensemble paradigm yielding the trained model B. These trained models can be used by the prediction system on the test feature to produce prediction labels. Finally, the performance can be evaluated by comparing predicted labels to gold-standard labels yielding performance parameters. The key benefit of using an ensemble classifier is its superior performance compared to either multiclass or multi-label strategies. The pseudo-code that represents the ensemble-based risk stratification process can be seen in Appendix C. The ensemble technique can be applied to the CVD field, as well as to other fields, such as education, Alzheimer’s, etc.

#### 4.3.1. Different Classifier Combination for Ensemble-Based CVD Risk Stratification

The different classifiers used in ensemble techniques were kNN, Reglog, GaussNB (GNB), linear discriminant analysis (LDA), quadratic discriminant analysis (QDA), random forest (RF) [91,95,96,97,98], multilayer perceptron (MLP), SVM [91,94,95,97,101,103,104], CNN, long short term memory network (LSTM), gated recurrent unit (GRU), bidirectional LSTM, bidirectional GRU [92], bagging, XGBoost, Adaboost [93,99], DNN [94], generalized additive models (GAMs), elastic net, penalized logistic regression (PLR), gradient boosted machines (GBMs), Bayesian logistic regression [96], K-NN [98,99,102,104,121], NB [101,104], light GBM, GBDT, LR, BPNN, DT [98,99,104,109], GB [99], Adaboost ensemble [100], ANN [101,104], GNB, LDA, LR, QDA, AdaBoost [105,113,118], XGBoost [102,118], ensemble SVM [104], CART [106], bagging, VS, LASSO, boosting, Bassian, MARS, logistic [107], ensemble boosting [80], ensemble learning, deep learning [108], ET, sequential minimal optimization (SMO), IBk, AdaBoostM1 with decision stump (DS), AdaBoostM1 with LR, REPTree, [109], neural network (NN), GB [110,114], linear Cox model [110], ensemble gradient boosting [111], ET [112], NB, multi-layer defense system (MLDS) [114], average- voting (AVEn), majority-voting (MVEn), weighted-average voting (WAVEn) [115], HTSA, ensemble deep learning [116], XGBoost Meta [117,119], SOM [120], extreme learning machine (ELM) [121].

#### 4.3.2. Comparison between the Three Types of CVD Risk Assessment Systems

All the architecture can be combined to achieve the functionality of all the three models, namely multiclass, multi-label [13], and ensemble. Both multiclass, multi-label modalities can be combined with the ensemble to acquire a better accuracy in the prediction of CVD risk. The comparison between the three has been shown in Appendix D, Table A1. The data size varies from 212–66,363 (for multiclass) [69,70,71,72,73,74,75,76,77,78,79,80,81,82], 300–46,520 (for multi-label) [83,84,85,86,87,88,89,90], 459–823,627 (for ensemble) [80,91,92,93,94,95,96,97,98,99,100,101,102,103,104,105,106,107,108,109,110,111,112,113,114,115,116,117,118,119,120,121]. The number of risk factors for multiclass is low, multi-label is more, and for the ensemble is moderate. The risk factors considered for multiclass are family history and BMI. For multi-label-based studies and ensemble-based studies, the risk factors considered were BMI, ethnicity, hypertension, and smoking. The image modalities used for multiclass and multi-label were MRI [175,176], ECG [177,178,179], and CUSIP whereas ECG is not used in ensemble-based studies. The range of performance evaluation parameters used for the multiclass, multi-label, and ensemble was 1–5, 1–8, and 1–8, respectively. The different types of classifiers used for these three techniques were SVM [91,94,95,97,101,103,104], RF [91,95,96,97,98], CNN, DT, k-NN, Agatston classifier, Elastic Net, NN, NB, XGBoost, SVM, ELM, one against one (OAO), one against all (OAA), decision direct acyclic graph (DDAG), exhaustive output error correction code (ECOC) [69,70,71,72,73,74,75,76,77,78,79,80,81,82]. The power analysis is also done on more multi-label and ensemble-based techniques. The detailed description can be seen in Appendix F. The general presentation of the NN algorithm was made in Appendix H.1 Right. The ML-based systems also lead to bias as it lacks clinical evaluation which is discussed in the next section. 

### 4.4. Performance Evaluation Metrics for Multiclass, Multi-Label, and Ensemble Techniques

**Figure 9:**
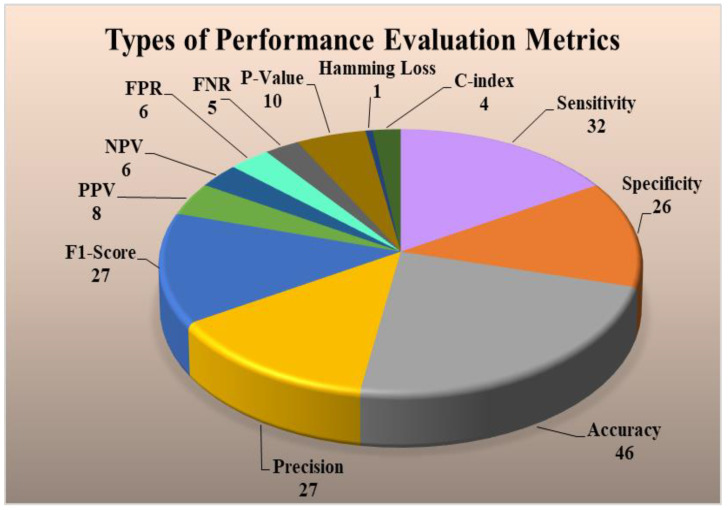

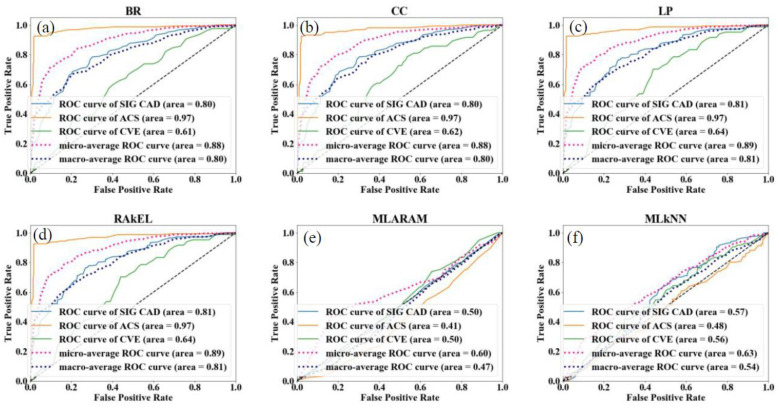
(**Top**) Types of performance evaluation metrics for ML-based CVD systems, (**Bottom**) Example of a ROC for multi-label-based CVD systems (Courtesy of AtheroPoint, Roseville, CA, USA) [84], PPV: positive predictive value; NPV: negative predictive value; FPR: false positive rate; FNR: false negative rate; BR: binary relevance; CC: classifier chain; LP: label powerset; MLARAM: multi-label adaptive resonance associative map; RakEL: random k-labelset; MLkNN: multi-label k-nearest neighbor; CVE: cardiovascular events; CAD: coronary artery disease; ACS: acute coronary syndrome; ROC: receiver operating characteristic; (**a**–**f**): different en-points used in the multi-label studies.

Performance evaluation (PE) strategies are very vital for understanding the reliability of the ML-based CVD risk stratification systems. The main metrics used by the PE systems are sensitivity, specificity, accuracy, precision, F1-score, positive predictive value (PPV), negative predictive value (NPV), false-positive rate (FPR), false-negative rate (FNR), *p*-value, hamming loss, C-index in multiclass, multi-label, and ensemble-based CVD risk assessment systems. The formulae used for determining these parameters are described in Appendix E. These different PE strategies were analyzed in different techniques. It was found that PE for multi-label-based CVD is different as compared to multiclass and ensemble. There are two types of PE techniques for multi-label, namely label-based and instance-based PE. The label-based is done using micro and macro-averaging techniques. Details of these techniques can be seen in Appendix E. Figure 9 (top) shows the label-based and instance-based performance evaluation. The number of studies that used this PE parameter is the accuracy (46) followed by sensitivity (32), precision (27), F1-score (27), specificity (26), *p*-value (10), PPV (8), NPV (6), FPR (6), FNR (5), c-index (4), Hamming Loss (1). Hamming Loss has opted only for the ensemble-based CVD risk stratification [181,182,183,184]. The PE metrics used in the stress test-based (ECG) [185,186,187] techniques are area-under-the-curve (AUC), sensitivity, specificity, PPV, and NPV [188,189,190,191,192].

As seen from the above discussion, the most important characteristic of the multiclass paradigm is the selection of gold standards having greater than two classes. The highest flexibility in the multiclass framework is the amalgamation of different sources of covariates, namely OBBM, LBBM, CUSIP, and MedUSE. We could take characteristics of plaque in the carotid ultrasound such as information about plaque symptomatology. The same principle holds in the stress test-based CVD paradigm or non-CVD framework. The ML systems sometimes overestimate the accuracies in prediction and underestimate the scientific validation, which results in bias in the prediction systems that we discuss in Section 5.

## 5. Bias Distribution in the ML System for Multiclass, Multi-Label, and Ensemble

**Figure 10:**
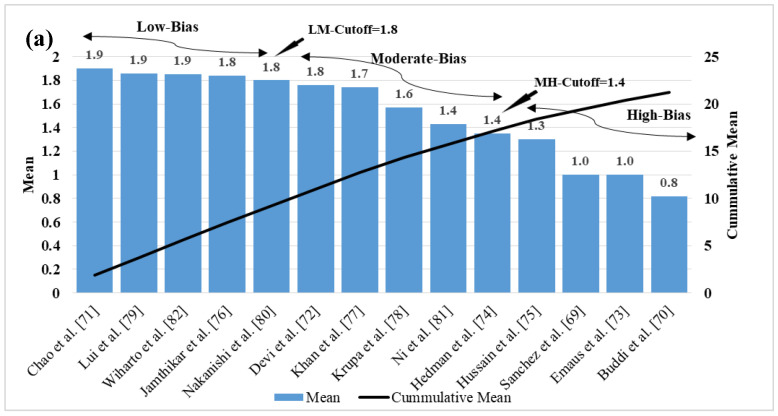

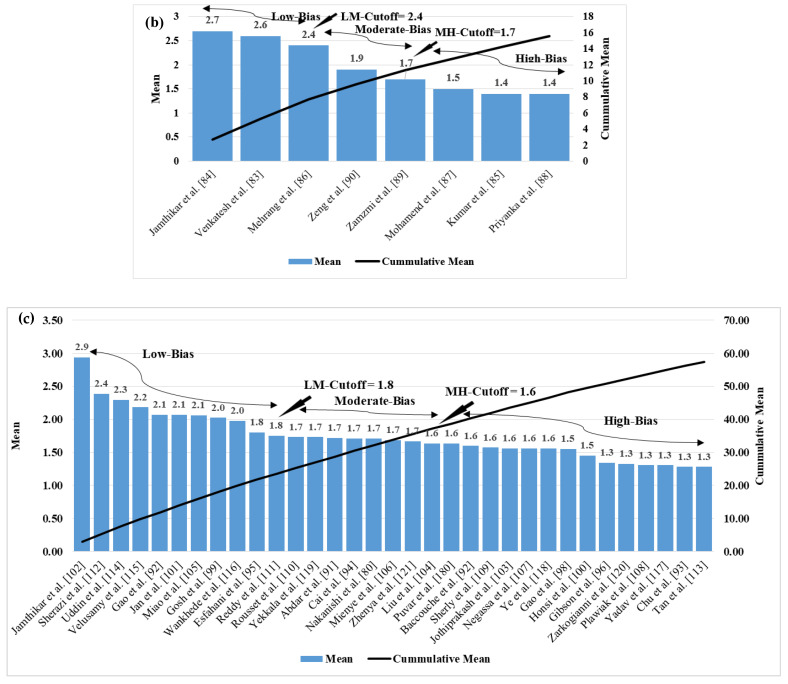

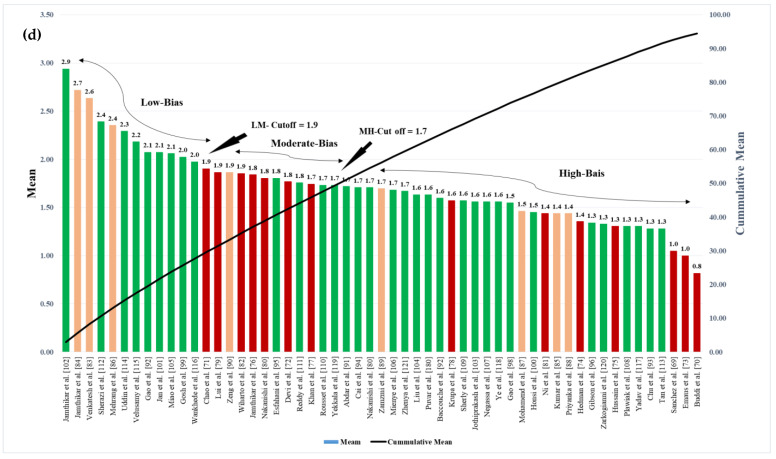
Cumulative plot for (**a**) multiclass studies (**b**) multi-label studies (**c**) ensemble studies (**d**) cumulative plot for all the ML studies.

The ML-based systems for CVD risk classification generate a bias due to various reasons [193,194,195]. Thus, it is important to understand the risk of bias (RoB) in these ML-based systems. As the ML systems were clustered in three different clusters, namely multiclass, multi-label, and ensemble, the bias nature was compared in three independent categories, and finally by considering all the three mixed together. For the RoB in the ML-based systems, the ML systems were ranked on the basis of the average mean score along with cumulative mean values (Table 5). The mean and the cumulative score were generated by scoring the ML attributes for each study. There were 52 ML studies (14 in multiclass, 8 in multi-label, 30 in ensemble cluster) with 41 attributes each. The score was given to each AI attribute using a grading scheme [196]. In this grading scheme, a high-score was assigned to the AI attribute, if the AI attribute was adopted (used) in a particular study (publication). The score is between 0 and 5. For example, a high-score was given if the attribute “data size” had a value higher than 1000 patients, else a low-score was assigned. Similarly, as another example, a high-score of 5 was given to the attribute “feature extraction”, if it was implemented in a study, else a score of 0 was assigned, if not implemented. Later the ML-based studies were clustered into low-bias, moderate-bias, and high-bias groups. The distributions were done on the basis of the two cut-offs values. The low-moderate (LM) and moderate-high (MH) cutoff values for each cluster of ML studies were determined based on the mean values along with the cumulative-mean values. The cutoffs values obtained for the multiclass cluster are 1.8 and 1.35 for LM and MH respectively (Figure 10a). The studies belonging in the low-bias, the moderate-bias, and the high-bias bins are 4, 5, and 5, respectively. Similarly, the cutoffs for the multi-label cluster are LM: 1.9 and MH: 1.4 (Figure 10b). Multi-label-based CVD ML studies in low-bias group are 3, moderate-bias group are 3 and high-bias group is 2. The values of LM cutoff for the ensemble cluster are 1.8 and HM cutoff value is 1.6. The studies in low-bias bin are 8, in moderate-bias are 16 and high-bias bin is 6 respectively for ensemble-based ML studies (Figure 10c). Alternatively, as all the studies are based on CVD risk prediction, the LM and MH cutoffs were determined by combining all the 52 studies. The LM, HM cutoff for the combined approach is 1.9 and 1.7 respectively (Figure 10d). Thus, we see that the ensemble-based ML CVD risk estimation systems are low-biased among all the selected studies followed by multiclass-based (moderate-biased) while the multi-label-based was found to be low-biased. The AI-based CVD risk stratification systems can be further improved by incorporating the mobile, cloud, and e-health infrastructure as discussed in the next Section 6.

## 6. CVD Risk Assessment through Mobile, E-Health, and Cloud Techniques

The CVD risk prediction was taken to next level by integration of mobile, cloud, and telemedicine technologies. The mobile-based CVD systems follow both ML and non-ML approaches [197,198,199,200,201,202,203,204,205,206,207]. The classifier techniques used for the mobile-based ML systems were k-NN [208], SVM [201,209], CNN [201,202], NB [204], DT [207], and RF [207]. The number of outcomes for the mobile-based CVD systems [197] varies between 1 and 2, basically CVD and diabetes. The cloud-based CVD systems also used both ML and non-ML approaches for CVD risk prediction [197,198,199,200,201,202,203,204,205,206,207,208,209]. The types of classifiers used for the cloud-based ML CVD risk prediction systems were quite similar to the mobile-based systems, namely SVM [201,209], k-NN [208], CNN [201,202], RF [207], Bayesian [204], and DT [207]. The number of outcomes changes to 1 in the cloud-based CVD systems [197,198,199,200,201,202,203,204,205,206,207,208,209]. All the mobile and cloud-based studies have performed the feature extraction along with the analysis for the CVD risk prediction. Cross-validation was also done by using the K-fold CV protocol (Column C17) for the mobile, as well as cloud-based systems [197,198,199,200,201,202,203,204,205,206,207,208,209]. For performance evaluation of the mobile and cloud-based CVD, systems were analyzed by the use of different parameters such as sensitivity [207,209], specificity [207,209], accuracy [207,209], precision, F1-Score, *p*-value, Silberg score [199], and receiver operating characteristic (ROC) [200] (Column C22–C29). However, the number of performance parameters used by each study ranges from 0 to 3 as described in Table A4.

Scientific validation (Column C12) was also performed for a high number of mobile and cloud-based CVD studies. Only one cloud-based CVD risk prediction system has been FDA approved (Column C6) [208]. All the characteristics are described in detail in Table A4. It can be noticed that the AI-based systems have gained the advantage of more accuracy, reliability with the addition of mobile and cloud-based infrastructure. It is also helpful in remote prediction, which is very much important in the COVID-19 framework. As the CVD prediction systems have evolved in the COVID-19 times, we, therefore, discuss this in the upcoming section.

## 7. Critical Discussion

### 7.1. Principal Findings

The main scope of this review was to compare comprehensively the three kinds of machine learning (ML) techniques mainly multiclass, multi-label, and ensemble in office-based settings. Further, the scope of the study had a limited discussion on (a) CVD risk prediction using ECG signals-based settings and (b) deep learning (DL) techniques for CVD risk prediction. Therefore, the main or principal findings from this review were (i) three types of CVD risk stratification techniques, namely (a) multiclass (b) multi-label, and (c) ensemble; (ii) types of covariates used where OBBM, LBBM, MedUSE, and CUSIP. The OBBM, LBBM, MedUSE were used widely when compared to image-based phenotypes (CUSIP), which is now evolving more rapidly since is a surrogate marker for coronary artery disease; (iii) ground truth is a very vital factor so as to avoid the risk of bias (RoB) during the ML-based CVD risk prediction; (iv) popularity of the classification techniques used in the field of CVD were in the order as multiclass-based, ensemble-based, multi-label-based; (v) clinical and scientific validation is another set of AI attributes that must be accompanied in any ML-based CVD risk prediction systems to prevent the AI bias from in such systems; (vi) the performance evaluation metrics used for the three techniques were analyzed. It was found that the most commonly used PE parameter was accuracy. The cloud-based AI techniques comprising all the three classifications techniques are more likely to be the future for CVD risk prediction. In the future, advanced computer-aided diagnosis techniques can be applied based on image processing [210]. Edge devices with mobile and cloud-based AI infrastructure are now highly emerging in the medical industry as it provides remote facility and is a much faster, the most necessary feature in the COVID-19 era.

### 7.2. Benchmarking Table

Table 6 shows the benchmarking table with a comparison between eighteen review studies that focused on multiclass, multi-label, and ensemble techniques for CVD risk prediction. This table shows thirteen attributes (column **C1** to column **C13**) for each of the eighteen studies [35,211,212,213,214,215,216,217,218,219,220,221,222,223,224,225,226] corresponding to the rows R1 to R18. These thirteen attributes presented were the Author (**C1**), year of the study (**C2**), name of the journal (**C3**), data size (**C4**), the study belongs to CVD or not (**C5**), the domain of the study (**C6**), machine learning (**C7**), classifier type (**C8**), cross-validation protocol (**C9**), the studies are multiclass (**C10**), multi-label study (**C11**), ensemble study (**C12**), and finally the summary of the study (**C13**). The data size for each study is shown in column **C4**, which is ranging from 8 to 86,155, whereas our study (row **R18**) has used 94 studies. Column **C5** describes whether the study is of CVD type or not. Studies (rows **R2**, **R3**, **R5**, **R9**, **R10**, **R11**, **R12**, **R16**, and **R17**) along with our study (row **R18**) are in the field of CVD while the rest are not. Column **C6** describes the different domains for the studies (rows **R1**, **R4**, **R6**, **R7**, **R8**, **R13**, **R14**, and **R15**) which does not belong to CVD. The domains are EEG, blood pressure, education, statistics, software, chronic fatigue, and sickle cells. The technical approach of the studies is shown in column **C7**, i.e., whether machine learning (ML) or not. Most of the studies including our proposed study are ML (rows **R1**, **R3**, **R4**, **R6**, **R7**, **R8**, **R9**, **R10**, **R11**, **R12**, **R13**, **R14**, **R15**, **R16**, **R17**, and **R18**). Column **C8** indicates the classifier types for the studies ranging from SVM, NN, LDA, OVO (row **R1**), RF, SVM, DT, KNN, LR, GNB (row **R3**), SMOTE (row **R4**) [227], Adaboost, KNN, BPSO (row **R6**), XG-Boost (row **R7**), RF, NBC, KNN (row **R8**), K-Star (row **R9**), SVM, RF, CNN (row **R10**), KNN, RF, DT (row **R11**), LDA (row **R13**), MULAN (row **R14**), LDA, MDDM (row **R15**), Probabilistic (row **R16**), LogitBoost (row **R17**). The cross-validation protocols used are shown in column **C9** which are K5 (rows **R3**, **R4**, **R17**), K7 (row **R6**), Open (row **R7**), K10 (rows **R8**, **R11**), and K* (row **R9**). The multiclass studies were (rows **R1**, **R3**, **R6**, **R7**, **R9**, **R11**, **R12**, **R17**) shown in column **C10** along with our study (row **R18**). Column **C11** shows multi-label studies (rows **R8**, **R13**, **R14**, **R15**, and **R18**) likewise column **C12** shows the ensemble studies (rows **R4**, **R6**, **R10**, and **R18**). The last column **C13** describes the keyword objectives of each study. The studies’ objectives were classification and CVD risk prediction or stratifications.

### 7.3. A Special Note on Non-Linear CVD Risk Stratification

The conventional classification CVD risk assessment systems assume the linear relationship between the covariates and the gold standard. The linear systems typically use the covariates like OBBM and LBBM or ECG signals [228,229,230]. With the additions of CUSIP and MedUSE, the requirement becomes more stringent on CVD calculators. In today’s times, it was observed that COVID-19 can play the role of a new covariate or risk factor due to its relationship with CVD [231,232]. The risk of CVD gets accelerated in the individual with COVID-19 [233,234]. This inclusion can result in a more non-linear classification paradigm for CVD risk prediction [235]. This can improve the reliability and the accuracy of the prediction results [236]. The AI/ML approaches help in understanding the non-linear relationship between the covariates and the ground truth. Hence there is a need for the development of non-linear classifiers in the ML/DL domain. It includes non-linear SVM classifiers [237], PCA, XGBoost [235], RF [233], generalized discriminant analysis (GDA), ELM, LDA [238]. Different non-linear methods which are applied in the CVD field are Poincare plot (PP), approximate entropy (ApEn) [235], quasi period density-prototype distance (QPD-PD) [239], fuzzy entropy [238], recurrence period density prototype distance (RPD-PD) [237], non-linear ensemble classifiers [233]. These are all out of the scope of the current study. The other application of non-linear classifiers are in the field of stroke [240] and sleep apnea [241]. The non-linearity can also be handled by using the DL approaches along with multiclass, multi-label, and ensemble-based techniques for CVD risk prediction in the future.

### 7.4. A Special Note on Time-to-Event for Cardiovascular Risk Prediction

This is one of the greatest assets of the machine learning system. The most important ingredient for accomplishing this solution is to ensure that we have a follow-up gold standard for the clinical data. This means one must have the gold standard (events) for the times such as 1st-year, 3rd-year, 5th-year, and 10th-year. Further, the risk factors (so-called covariates or variables) must be available for the development of the training model. Given the two pairs (covariates and the gold standard-even for that time), one can develop the machine learning model for that time-zone (1st-year, 3rd-year, 5th-year, and 10th-year). Should you intended to predict for 1st-year, 3rd-year, 5th-year, and 10th-years, it requires four kinds of machine learning models. Each time-event has to have its own machine learning model. The atherosclerosis disease which has transformed over different years and leads to the event needs to be used for the development of the training model. The only challenge with this setup is the length of time it takes to collect the event data. It is both expensive and tedious since we have to follow the patients over the 10-year period. Recently, Kakadiaris et al. [62] perused this strategy using the machine learning paradigm. The ML paradigm has the same fundamental concept of training and testing as shown in Figure 4. The left half is the training model where the gold standard will change as per the time-zone (1st-year, 3rd-year, 5th-year, and 10th-years), while the prediction will be applied for the patient for the corresponding time-zones (1st-year, 3rd-year, 5th-year, and 10th-years). It is painful to wait to accomplish this validation, since it is costly, and a large cohort is needed.

To overcome such a scenario, another way to predict the CVD risk is using the surrogate marker of carotid artery disease. Since the formation of the atherosclerotic disease in coronary artery has the same genetic make-up as the carotid artery disease, the surrogate artery can be used for the prediction of CVD or the coronary artery disease risk. Further, note that over time (1st-year, 3rd-year, 5th-year, and 10th-years), the plaque formation changes and so does the image phenotypes such as intima-media thickness, plaque burden, or plaque area/volume. Thus, one can compute the time-dependent image phenotypes which uses the ingredients which make the atherosclerotic disease. This includes rate of change of cIMT over time (age), obesity index over time (age), cholesterol change over (age), one can use this paradigm to predict the plaque burden in carotid artery-based age. This is sometimes called as vascular age of the patient. This has been shown by Khanna et al. [34]. Later, this was commercialized as AtheroEdge™ 2.0 (AtheroPoint™, Roseville, CA, USA) [36]. The CVD risk can be computed based on the intensity of the risk factors. This is called a non-ML method (also known as the statistical solution for the prediction of the 10th-year CVD risk.

### 7.5. A Special Note on the Advantages of Machine Learning-Based Cardiovascular Risk Stratification

Machine learning paradigm for CVD risk prediction has provided us with a way to obtain more accurate, early, and fast results. The ML systems offer following advantages against the previously published studies: (i) it handles the non-linear nature between the covariates and ground truths (GT) [31]; (ii) ability to predict the CVD risk in granular classes, such as six different risk classes (no-risk, low-risk, mild-risk, moderate-risk, high-risk, and very-high-risk) [34,35]; (iii) ability to augment the training data using popular augmentation paradigms such as adaptive synthetic (ADASYN) and synthetic minority over-sampling technique SMOTE [227]; (iv) incorporate the cohort’s knowledge during training and predicting the CVD risk; (v) flexibility of amalgamating of different types of covariates such as OBBM, LBBM, CUSIP, and MedUSE during the design of the model training; (vi) ability to interface with different types of classification techniques like multiclass, multi-label and ensemble for improving the overall performance of the system; and (vii) ability to enhance the risk factor (or covariates) such as genetic and comorbidities such as cancer. Thus, all the above-mentioned factors puts ML-based system a very strong paradigm for CVD risk stratification, unlike the conventional statistical models.

### 7.6. A Special Note on Deep Learning-Based Cardiovascular Risk Stratification

The Deep learning (DL) paradigm has started to emerge in the field of CVD risk prediction. The DL approach can be applied for both (a) the office-based [242,243] and (b) stress-based test settings [244,245,246,247,248]. DL approaches have been applied for CVD risk stratification using multiclass [249], multi-label [250], and ensemble-based paradigms [116]. Even though there are evolving CVD risk stratification techniques in the DL framework, this review does not venture deep since it is not the main focus of this review. As a result, we have not analyzed publications related to the DL paradigm. Note that, the main advantage of DL techniques is (i) automated feature selection process from the input covariates (such as OBBM, LBBM, CUSIP, and EGC signals phenotype) and (ii) prediction of more accurate and reliable results due to a large number of layers in DL network. Advanced stochastic imaging methods can be applied [251] to improve the loss function during the training paradigm. This evolving DL paradigm will flourish more in the very near future in office-based imaging and stress-based test settings.

### 7.7. The Future of Cardiovascular Disease Risk Stratification

The CVD risk estimation at an early stage is very much important to reduce the mortality rate due to CVD [252,253]. As it was observed that not only ML but extreme machine learning (ELM) can also be applied and further developed for CVD risk stratification [254]. Moreover, COVID-19 accelerates the atherosclerosis condition due to which fast detection of CVD in COVID-19 patients is needed [255,256]. The above circumstances are leading to an evaluation in the CVD risk stratification techniques. In the near future, cloud-based AI modalities will be very much in use for CVD risk detection. It also promotes the remote and fast prediction of the risk of CVD. It also helps in reducing prediction errors. Other non-invasive imaging techniques like carotid, femoral, arterial imaging can be used as an indirect measure of plaque build-up in these arteries. Deep learning technologies will evolve in the field of CVD risk estimation [257]. This will also include pruning of weights using evolutionary techniques such as genetic algorithms in the Deep Learning framework [147]. Devices equipped with cutting edge technologies like mobile-based AI, cloud-based AI, multiclass, multi-label, and ensemble-based systems for CVD risk prediction will be emerging in the medical imaging industry market.

## 8. Conclusions

This was the first review study of its kind that presented three different kinds of AI-based CVD risk stratification, namely multiclass, multi-label, and ensemble, where multiclass was most popular and multi-label was least, which happened to be our first key contribution. The second contribution was exhaustive analysis by selecting the best 265 studies using the PRISMA model for understanding the three kinds of machine learning-based systems for prediction of the CVD risk. This was based on our hypothesis that there exists a biological link between atherosclerotic disease formation and the CVD risk. The third contribution was the identification of the top four covariates, namely OBBM, LBBM, CUSIP, and MedUSE for designing the training model using a machine learning framework. The fourth contribution was on the choice of the gold standard for an unbiased AI system design for CVD risk prediction, which leads to a robust and reliable CVD prediction system. The fifth finding and contribution required that the ML system undergo clinical and scientific validation for reliability, stability, and robustness of the system design. Lastly, we observed that with the advancement of telecommunication systems, mobile and cloud-based strategies are speedily penetrating the CVD risk stratification system designs. Low-powered edge devices like Rasberry Pi and Jetsen Nano are like to be adopted in the future.

## Figures and Tables

**Table 1:** Multiclass 14 CVD studies and their characteristics in ML/DL framework.

SN	Studies	Input Covariates	Gold Standard Types	#RC	ML/DL
1	Chao et al. [71]	OBBM, LBBM	CVD Event	K	DL
2	Lui et al. [79]	ECG parameters	CHC	3	ML
3	Wiharto et al. [82]	OBBM, LBBM, ECG	CHD	3	ML
4	Jamthikar et al. [76]	OBBM, LBBM, CUSIP	CVE	4	ML
5	Nakanishi et al. [80]	OBBM, LBBM, CUSIP	Death	3	ML
6	Devi et al. [72]	ECG Parameters	SCD	3	ML
7	Khan et al. [77]	PCG Signals	CVE	3	ML
8	Krupa et al. [78]	APG signals	BCVD	3	ML
9	Ni et al. [81]	ECG Signals	CVD, No CVD	4	DL
10	Hedman et al. [74]	OBBM, LBBM	Heart Failure	3	ML
11	Hussain et al. [75]	OBBM, LBBM, ECG	MI	3	ML
12	Sanchez et al. [69]	OBBM, LBBM	CAC score	9	ML
13	Emaus et al. [73]	OBBM, CAC (CT)	F/NF CVD	3	DL
14	Buddi et al. [70]	OBBM, LBBM	CVD, Diabetes	4	ML

SN: Serial number; APG: Acceleration plethysmogram; CHD: Coronary heart disease; CVE: Cardiovascular events; CHC: Chronic heart conditions; SCD: Sudden cardiac death; BCVD: Binary CVD (Healthy, diseased); F/NF CVD: Fetal/Non-fetal CVD; CT: Computed tomography; #RC: Risk classes; OBBM: Office-based biomarkers; LBBM: Laboratory-based biomarkers; CUSIP: Carotid ultrasound image phenotypes; CAC: Coronary artery calcium; ECG: Electrocardiogram; MI: Myocardial infarction.

**Table 2:** Multiclass in CVD vs. non-CVD using seven attributes.

SN	Attributes	Multiclass CVD	Multiclass Non-CVD
1	Ground truth types	CVE [69,70,71,72,73,76,77,78,79,81,82], HF [74], MI [75], Death [80]	AD, NC, MCI, PMCI vs. SMCI [141], Proliferation, NP [139], ADH, DCS, IC [137,138,142]
2	Covariates types for the ML design	OBBM [69,70,73,74,75,76,80,82], LBBM [69,70,73,74,75,76,80,82], CUSIP [71,72,76,77,78,79,80,81,82], MU [76]	BHI [139], OBBM [137,138,141,142], LBBM [137,138,141,142]
3	Disease Type	CVD [69,70,71,72,73,74,75,76,77,78,79,80,81,82]	Diabetes [142], Cancer (Breast, Lung, Brain) [138,139], Alzheimer’s [138,141], Retinal [137]
4	Image Modalities	ECG, CT, US [71,72,76,77,78,79,80,81,82]	EEG, MRI, CT [137,139]
5	# Classes	3–9 [69,70,71,72,73,74,75,76,77,78,79,80,81,82]	5–14 [137,138,139,141,142]
6	Architecture Type	ML [70,72,76,77,78,79,80,82], DL [71,81]	ML, rMLTFL [141]
7	Classifiers used	SVM [70,75,76,77], DT, RF, LR, NB, KNN, CNN [71,79]	RetiCAC [137], PCE, SVM, CNN, DT, LR, NB, SVM, KNN, ensemble [138,139]

SN: Serial number; CVE: Cardiovascular event; AD: Alzheimer’s; NC: Normal control; MCI: Mild Cognitive impairment; PMCI: progressive MCI; SCMI: Significant memory concern; HF: Heart failure; MI: Myocardial infraction; OBBM: Office-based biomarkers; LBBM: Laboratory-based biomarkers; CUSIP: Carotid ultrasound image phenotype; ECG: Electrocardiogram; CT: Computed tomography; US: Ultrasound; MRI: Magnetic resonance imaging; BHI: Breast histopathology images; MU: MedUse; IM: Image modalities; SVM: Support vector machine; KNN: K-nearest neighbor; DT: Decision tree; RF: Random forest; LD: Logistic regression; NB: Naive Bayesian. RetiCAC: Deep learning retinal CAC score; PCE: Pooled cohort equation; rMLTFL: robust multi-label transfer feature learning.

**Table 3:** Multi-label 8 studies and their characteristics.

SN	Studies	Input Covariates	Ground Truth	ML/DL
1	Venkatesh et al. [83]	OBBM, LBBM	Death, Stroke, CHD, CVD, HF, AF	ML
2	Jamthikar et al. [84]	OBBM, LBBM, CUSIP	CAD, ACS, Composite CVE	ML
3	Kumar et al. [85]	OBBM, LBBM, ECG	LVD, SVD, ICH	ML
4	Mehrang et al. [86]	OBBM, LBBM, CUSIP	Non-AFib-Non-ADHF, Afib-Non-ADHF, Afib-ADHF	ML
5	Mohamend et al. [87]	OBBM, LBBM, CUSIP	SHF, ASHF, CSHF, ACSHF, DHF, ADHF, CDHF, ACDHF	ML
6	Priyanka et al. [88]	OBBM, LBBM	HT, CHF, AF, CA, AKF, Dia-TII, HL, ARF, UTI, ER	ML
7	Zamzmi et al. [89]	MRI, CT Signals	HF, CAD, DCM, MI	DL
8	Zeng et al. [90]	OBBM, LBBM	LC, CC, IC, RC	ML

SN: Serial number; HF: Heart failure; AF: Arterial fibrillation; LVD; Large vessel disease; SVD: Small vessel disease; ICH: Intracerebral hemorrhage (ICH); SHF: Systolic heart failure; ASHF: Acute systolic heart failure; CSHF: Chronic systolic heart failure; ACSHF: Acute on chronic systolic heart failure; DHF: Diastolic heart failure; ADHF: Acute diastolic heart failure; CDHF: Chronic diastolic heart failure; ACDHF: Acute on chronic diastolic heart failure; HT: Hypertension; CHF: Congestive heart failure; CA: Coronary atherosclerosis, AKF: Acute kidney failure; HL: Hyperlipidemia; Dia-TII: Diabetes Type II; ARF: Acute respiratory failure; UTI: Urinary tract infection; ER: Esophageal reflux; DCM: Dilated cardiomyopathy LC: Lung complication, CC: Cardiac complication; IC: Infectious complication, RC: Rhythmia complication.

**Table 4:** Ensemble-based 33 and their characteristics of ML-based.

SN	Studies	Input Covariates	Ground Truth	ML/DL
1	Abdar et al. [91]	OBBM, LBBM	CAD	ML
2	Baccouche et al. [92]	OBBM, LBBM	HHD, IHD, MHD, VHD	DL
3	Chu et al. [93]	OBBM, LBBM, ECG	CVD, Dia	ML
4	Cai et al. [94]	OBBM, LBBM	CR	ML
5	Esfahani et al. [95]	OBBM, LBBM	CVD	ML
6	Gibson et al. [96]	OBBM, LBBM	ACS	ML
7	Gao et al. [97]	OBBM, LBBM, ECG	CVD, BC	ML
8	Gao et al. [98]	OBBM, LBBM	CVD	ML
9	Gosh et al. [99]	OBBM, LBBM, ECG	CVD	ML
10	Honsi et al. [100]	OBBM, LBBM	CVD	ML
11	Jan et al. [101]	OBBM, LBBM, ECG	HD	ML
12	Jamthikar et al. [102]	OBBM, LBBM, CUSIP	CAD, ACS	ML
13	Jothiprakash et al. [103]	OBBM, LBBM	CVD	ML
14	Liu et al. [104]	OBBM, LBBM	CA	ML
15	Miao et al. [105]	OBBM, LBBM, ECG	CHD	ML
16	Mienye et al. [106]	OBBM, LBBM	HD	ML
17	Negassa et al. [107]	OBBM, LBBM	HF	ML
18	Nakanishi et al. [80]	OBBM, LBBM, CT	Death	ML
19	Plawiak et al. [108]	OBBM, LBBM, ECG	Arrhythmia	DL
20	Puvar et al. [180]	OBBM, LBBM, ECG	HD	ML
21	Reddy et al. [109]	OBBM, LBBM	HD	ML
22	Rousset et al. [110]	OBBM, LBBM	CVD	ML
23	Sherly et al. [111]	OBBM, LBBM, ECG	HD	ML
24	Sherazi et al. [112]	OBBM, LBBM	CVE	ML
25	Tan et al. [113]	OBBM, LBBM	CVD	ML
26	Uddin et al. [114]	OBBM, LBBM	CVD	ML
27	Velusamy et al. [115]	OBBM, LBBM	CAD	ML
28	Wankhede et al. [116]	OBBM, LBBM	HD	DL
29	Yadav et al. [117]	OBBM, LBBM	HD	ML
30	Ye et al. [118]	OBBM, LBBM	HYT	ML
31	Yekkala et al. [119]	OBBM, LBBM	CVD	ML
32	Zarkogianni et al. [120]	OBBM, LBBM	CVD, Dia	ML
33	Zhenya et al. [121]	OBBM, LBBM, ECG	HD	ML

SN: Serial number; HHR: Hypertensive heart disease; IHD: Ischemic heart disease, MHD: Mixed heart disease; VHD: Valvular heart disease; CR: Cardiac resynchronization; ACS: Acute coronary syndrome; CVD: Cardiovascular disease; CA: Cardiac arrhythmia; BC: Breast cancer; HD: Heart disease; HF: Heart failure; CVE: Cardiovascular event; Dia: Diabetes.

**Table 5:** Ranking table (**a**) multiclass studies, (**b**) multi-label studies, (**c**) ensemble studies.

**(a) Multiclass Studies**	**Sum**	**Mean**	**Rank**	**(c) Ensemble Studies**	**Sum**	**Mean**	**Rank**
Chao et al. [71]	78	1.9	1	Jamthikar et al. [102]	120.5	2.9	1
Lui et al. [79]	76.5	1.9	2	Sherazi et al. [112]	98	2.4	2
Wiharto et al. [82]	76	1.9	3	Uddin et al. [114]	94	2.3	3
Jamthikar et al. [76]	75.5	1.8	4	Velusamy et al. [115]	89.5	2.2	4
Nakanishi et al. [80]	74	1.8	5	Gao et al. [97]	85	2.1	5
Devi et al. [72]	72.5	1.8	6	Jan et al. [101]	85	2.1	6
Khan et al. [77]	71.5	1.7	7	Miao et al. [105]	84.5	2.1	7
Krupa et al. [78]	64.5	1.6	8	Gosh et al. [99]	83	2	8
Ni et al. [81]	59	1.4	9	Wankhede et al. [116]	81	2	9
Hedman et al. [74]	55.5	1.4	10	Esfahani et al. [95]	74	1.8	10
Hussain et al. [75]	53.5	1.3	11	Reddy et al. [111]	72	1.8	11
Sanchez et al. [69]	43	1	12	Rousset et al. [110]	71	1.7	12
Emaus et al. [73]	41	1	13	Yekkala et al. [119]	71	1.7	13
Buddi et al. [70]	33.5	0.8	14	Abdar et al. [91]	70.5	1.7	14
**(b) Multi-label Studies**	**Sum**	**Mean**	**Rank**	Cai et al. [94]	70	1.7	15
Jamthikar et al. [84]	111.5	2.7	1	Nakanishi et al. [80]	70	1.7	16
Venkatesh et al. [83]	108	2.6	2	Mienye et al. [106]	69	1.7	17
Mehrang et al. [86]	96.5	2.4	3	Zhenya et al. [121]	68.5	1.7	18
Zeng et al. [90]	76.5	1.9	4	Liu et al. [104]	67	1.6	19
Zamzmi et al. [89]	69.5	1.7	5	Puvar et al. [180]	67	1.6	20
Mohamend et al. [87]	60	1.5	6	Baccouche et al. [92]	65.5	1.6	21
Kumar et al. [85]	59	1.4	7	Sherly et al. [109]	64.5	1.6	22
Priyanka et al. [88]	59	1.4	8	Jothiprakash et al. [103]	64	1.6	23
				Negassa et al. [107]	64	1.6	24
				Ye et al. [118]	64	1.6	25
				Gao et al. [98]	63.5	1.5	26
				Honsi et al. [100]	59.5	1.5	27
				Gibson et al. [96]	55	1.3	28
				Zarkogianni et al. [120]	54.5	1.3	29
				Plawiak et al. [108]	53.5	1.3	30
				Yadav et al. [117]	53.5	1.3	31
				Chu et al. [93]	52.5	1.2	32
				Tan et al. [113]	52.5	1.2	33

**Table 6:** Benchmarking table for the multiclass, multi-label, and ensemble studies in CVD/non-CVD field.

	C1	C2	C3	C4	C5	C6	C7	C8	C9	C10	C11	C12	C13
SN	Author	Yr	JOU	DS	CVD	Domain	ML	CT	CVP	MC	MLB	Ensbl	Summary
**R1**	Boernama et al. [211]	**’21**	IEEE	30	🗶	EEG	🗸	SVM, NN, LDA, OVO	🗶	🗸	🗶	🗶	EEG Classification
**R2**	Collins et al. [212]	**’16**	BMJ	122	🗸	BP	🗶	🗶	🗶	🗶	🗶	🗶	CVD Meta-analysis
**R3**	Dissanayake et al. [213]	**’21**	Hindawi	CHDD	🗸	🗶	🗸	RF, SVM, DT, KNN, LR, GNB	K5	🗸	🗶	🗶	CVD risk
**R4**	Galar et al. [214]	**’12**	IEEE Tran.	Imb D	🗶	🗶	🗸	SMOTE	K5	🗶	🗶	🗸	Ensemble Classification
**R5**	Stewart et al. [215]	**’17**	JRSMCD	🗶	🗸	🗶	🗶	🗶	🗶	🗶	🗶	🗶	CVD risk
**R6**	Mathew et al. [216]	**’21**	IEEE	6	🗶	Edu	🗸	Adaboost, KNN, BPSO	K7	🗸	🗶	🗸	Teaching Quality
**R7**	Uike et al. [217]	**’21**	IEEE	8	🗶	SC	🗸	XG-Boost	Open	🗸	🗶	🗶	SC Classification
**R8**	Wang et al. [218]	**’14**	Plos One	736	🗶	CF	🗸	RF, NBC, KNN	K10	🗶	🗸	🗶	CF Classification
**R9**	Wiharto et al. [219]	**’16**	HIR	303	🗸	🗶	🗸	K-Star	K*	🗸	🗶	🗶	CHD Classification
**R10**	Boi et al. [220]	**’18**	CAR	126	🗸	🗶	🗸	SVM, RF, CNN	🗶	🗶	🗶	🗸	OCT-based risk stratification
**R11**	Jamthikar et al. [35]	**’20**	CBM	208	🗸	🗶	🗸	KNN, RF, DT	K10	🗸	🗶	🗶	CVD risk
**R12**	Bianchini et al. [221]	**’08**	IEEE	10	🗸	🗶	🗸	🗶	🗶	🗸	🗶	🗶	Cardiovascular Risk Markers
**R13**	Liu et al. [222]	**’12**	IEEE	15	🗶	Statistics	🗸	LDA	🗶	🗶	🗸	🗶	Statistical Classification
**R14**	Charte et al. [223]	**’20**	IEEE	🗶	🗶	Software	🗸	MULAN	🗶	🗶	🗸	🗶	Comparison
**R15**	Siblini et al. [224]	**’15**	IEEE	156	🗶	DM	🗸	LDA, MDDM	🗶	🗶	🗸	🗶	DM Reduction
**R16**	Indhumathi et al. [225]	**’21**	IEEE	30	🗸	🗶	🗸	Probabilistic	🗶	🗶	🗶	🗶	CVD Management
**R17**	Kolli et al. [226]	**’19**	IEEE	86,155	🗸	🗶	🗸	LogitBoost	K5	🗸	🗶	🗸	Coronary Artery Calcification
**R18**	Proposed Study	**’22**	🗶	265	🗸	🗶	🗸	🗶	🗶	🗸	🗸	🗸	CVD risk

DS: Data size; ML: Machine learning; CVP: Cross-validation protocol; MC: Multiclass; MLB: Multi-label; GNB: Gaussian I Bayes; HD: Heart disease; CHDD: Cleveland heart disease datasets; Ensbl: Ensemble; IEEE Tran: IEEE Transaction; JRSMCD: Journal of the Royal Society of Medicine Cardiovascular disease; CT: Classifier type; ImbD: Imbalance data; JOU: Journal; SC: Sickle cells; CF: Chronic fatigue.

**Figure A1:**
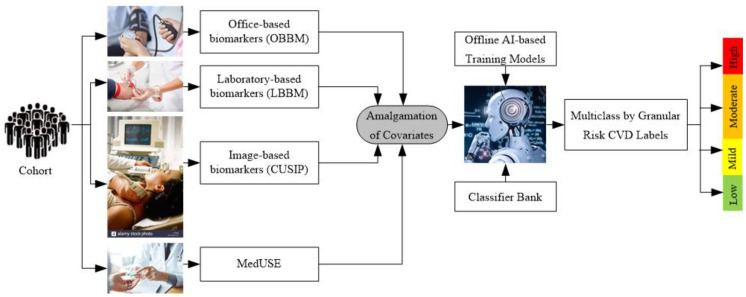
Typical online system for multiclass CVD risk stratification.

**Figure A2:**
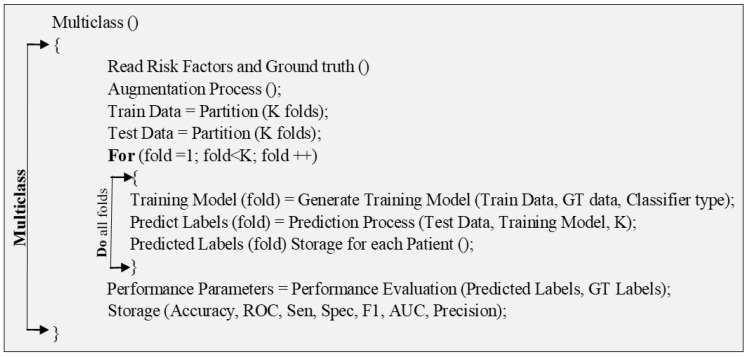
Pseudo-code for multiclass technique.

**Figure A3:**
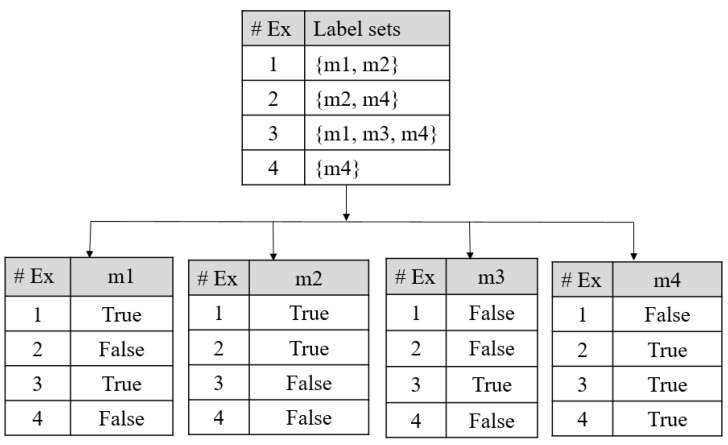
BR classifier, #Ex: Example.

**Figure A4:**
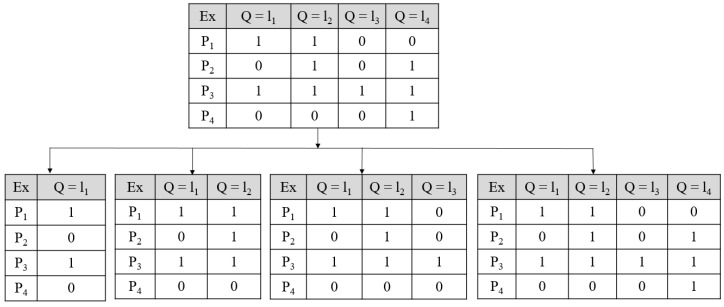
Classifier chain functioning; P_1_–P_4_ are data points under; Ex; Ex: Example.

**Figure A5:**
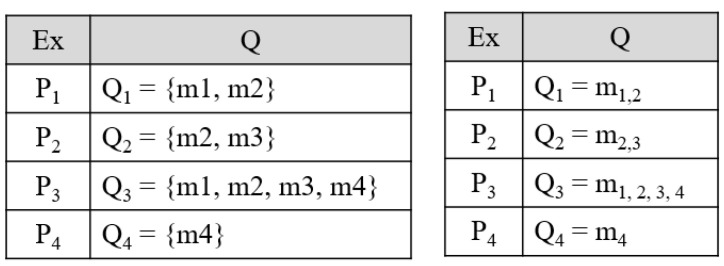
Label powerset transformation, Ex: Example.

**Figure A6:**
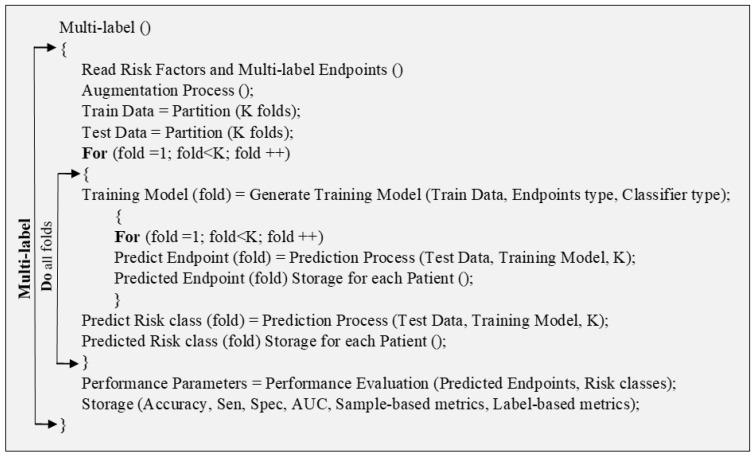
Pseudo-code for multi-label technique.

**Figure A7:**
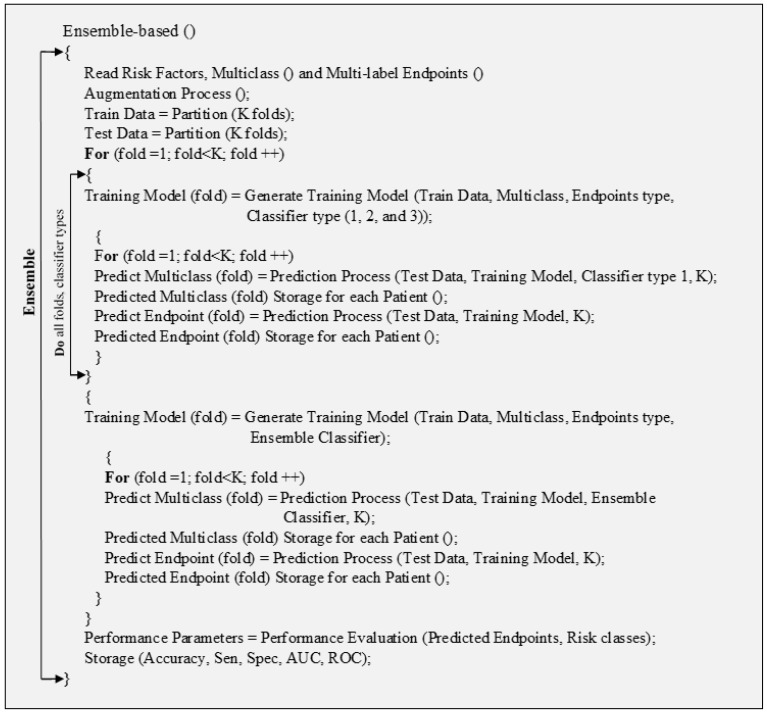
Pseudo-code for ensemble-based technique.

## Data Availability

Not applicable.

## Appendix A. Pseudo-Code for Multiclass Classification

### Appendix A.1. Typical Online System for CVD Risk Stratification for Multiclass

This system shows the amalgamation of online covariates, which are then transformed by the ML-based training model using multiclass-based models. The output yields the multiclass risk marked in color (low, mild, moderate, and high risk).

### Appendix A.2. Pseudo-Code for Multiclass

The pseudo-code describes the process used by the multiclass algorithm for CVD risk stratification into granular risk classes. It uses the “for” loop for training and prediction of each fold of data, which were divided into K folds. The training model is applied to the test data and the PE was predicted and stored in form of accuracy (ACC), ROC, sensitivity (Sen), specificity (Spec), F1-score, the area-under-the-curve (AUC), and precision.

## Appendix B. Pseudo-Code for Multi-Label Classification

### Appendix B.1. Problem Transformation Methods for Multi-Label Prediction

The problem transformation method (PTM) makes the multi-label classification problem to one or more single label classification tasks. Basically, four PTM, namely BR, CC, LP, and RakEL were used as discussed below:

*Binary Relevance:* In the BR technique, the problems get divided into one or more single-label classification problems. The single-label classification resembles the binary prediction. An example can be described as, say M is a set of “q” labels with M = {m1, m2, …, Mq}, the BR technique makes “q” single-label binary classifiers for each label. The multi-label training sets get converted to binary datasets (“q”), and Elj = 1…q, where Elj has all samples of the original dataset but with single positive or negative values. The dataset gets divided into “q” single label datasets with classifier C and the next classifier set is obtained as Cj (E), j = 1…q by the training set Elj. The label dependency was not considered by the BR classification algorithm. Thus, it shows less complexity in the computation as compared with other multi-label techniques. The process is shown in the following Figure A3 [258]. As shown in Figure A3 four examples were considered as multi-label dataset and label set M with four labels (m1, m2, m3, and m4) which is split as four single labels that are independent.

*Classifier Chain*: This algorithm also works in single-label classification. This technique takes a class of classifiers where the very initial classifier is trained with the dataset, which acts as the input, following that each classifier gets trained with the whole feature space. The feature set has an original dataset with the label set used in the earlier base classifier that is in the chain. Each base classifier uses the earlier label information for training and testing models. Thus, a correlation exists in the CC algorithm. Figure A4 describes the functioning of CC [259].

*Label Powerset*: It also converts the prediction situation to a single-label multiclass prediction technique. In this technique, all possible individual group of labels is given special or unique class. Such as if three types of labels are there, then eight different types of combinations can come into the picture. LP technique has eight types of labels that get trained for prediction. This technique deals with a large number of classes that are related to small instances, and also consideration of correlation is done. The transformation was shown in Figure A5 [260]. In Figure A5 the 1st table shows the original datasets, and the 2nd table is showings the transformed datasets.

*Random k-label set*: It is a type of combination technique used for multi-label prediction. Every combination method gets trained on a small size of the randomly selected subset of labels by a single-label-based classifier. This process is described as if L labels in the dataset (E), the RAkEL classifier turns this data to all the possible k-label sets (L_k_). Each label set is then trained for prediction. Finally, the prediction is made into positive (1) and negative (0) values in accordance with the threshold (0.5). The further implementation can be seen in [261].

### Appendix B.2. Algorithm Adaptation Methods for Multi-Label Prediction

*Multi-label KNN*: This algorithm is basically an implementation of the KNN algorithm in multi-label datasets. The neighbors are selected from unseen training sets. Next, the labelset are found for the instance which are unseen in nature by utilizing the maximum of posteriori (MAP) principle. The full algorithm can be seen in [262].

*Multi-label ARAM*: It is associated with the neural network model based on resonance theory. The advantage of this algorithm is its fast learning ability. The detailed algorithm can be seen in [263].

### Appendix B.3. Pseudo-Code for Multi-Label Classification Technique

Multi-label pseudo-code describes the multi-label algorithm where more than one multi-label endpoint was considered. For each multi-label endpoint, the risk class was defined. In this pseudo-code, two “for” loops are used one for multi-label and the next for multiclass prediction. Finally, the PE was determined as accuracy, sensitivity (Sen), specificity (Spec), area-under-the-curve (AUC), sample-based, and label-based metrics.

## Appendix C. Pseudo-Code for Ensemble Classification

### Pseudo-Code for Ensemble-Based Technique

Ensemble-based-CVD risk prediction uses combinations of multiple classifiers. The pseudo-code shows that the data are divided into testing and training with K folds. The prediction was done using each type of classifier for multiclass and multi-label prediction. Then each type of classifier is combined into an ensemble classifier and the final prediction was made.

## Appendix D. Comparison between 3 Paradigms

### Comparison of ML-Based Multiclass, Multi-Label, and Ensemble CVD Classification

**Table A1:** Comparison of ML-based multiclass, multi-label, and ensemble CVD classification.

**SN**	**Attributes**	**Multiclass**		**Multi-Label**		**Ensemble**	
**-**	**-**	**Characteristics**		**Characteristics**		**Characteristics**	
	Total Studies	14	[69,70,71,72,73,74,75,76,77,78,79,80,81,82]	8	[83,84,85,86,87,88,89,90]	32 [80,91,92,93,94,95,96,97,98,99,100,101,102,103,104,105,106,107,108,109,110,111,112,113,114,115,116,117,118,119,120,121]	
1	Data Size	212–66,363	[69,70,71,72,73,74,75,76,77,78,79,80,81,82]	300–46,520	[83,84,85,86,87,88,89,90]	459–823,627 [80,91,92,93,94,95,96,97,98,99,100,101,102,103,104,105,106,107,108,109,110,111,112,113,114,115,116,117,118,119,120,121]	
2	Risk Factors	Low	[69,70,71,72,73,74,75,76,77,78,79,80,81,82]	Large	[83,84,85,86,87,88,89,90]	Moderate [80,91,92,93,94,95,96,97,98,99,100,101,102,103,104,105,106,107,108,109,110,111,112,113,114,115,116,117,118,119,120,121]	
3	Family History	Frequent Considered	[69,71,76,77,80,82]	Seldom Considered	[83,84,90]	Considered Intermittently [80,91,96,97,99,100,102,105,106,110,111,112,114,115,116,117,118,119,120]	
4	BMI	Less considered	[72,74,75,76,80]	Considered Moderately	[84,85,86]	Highly considered [46,47,48,49,50,51,52,80,91,93,94,95,96,97,99,100,102,106,107,112]	
5	Ethnicity	Less Considered	[72,74,75,76,80]	Considered Moderately	[84,85,86]	Highly Considered	
6	Type of data	OBBM and LBBM	[69,70,71,72,73,74,75,76,77,78,79,80,81,82]	OBBM, LBBM and Image	[83,84,85,86,87,88,89,90]	OBBM and LBBM [80,91,92,93,94,95,96,97,98,99,100,101,102,103,104,105,106,107,108,109,110,111,112,113,114,115,116,117,118,119,120,121]	
7	Hypertension	Low Usage	[72,74,75,76,80]	High Usage	[83,84,85,86,87,88,89,90]	Moderate Usage [46,47,48,49,50,51,52,80,91,93,94,95,96,97,99,100,102,106,107,112]	
8	Smoking	Low Usage	[72,74,75,76,80]	High Usage	[83,84,85,86,87,88,89,90]	Moderate Usage [80,91,96,97,99,100,102,105,106,110,111,112,114,115,116,117,118,119,120]	
9	Multicenter	Low Usage	[72,74,75,76,80]	High Usage	[83,84,85,86,87,88,89,90]	Moderate Usage [80,91,96,97,99,100,102,105,106,110,111,112,114,115,116,117,118,119,120]	
10	MRI	Considered Moderately	[71,80]	Considered Moderately	[83,89]	Less Considered [80]	
11	ECG	Partial Considered	[72,74,75,78,79,81,82]	Strongly Considered	[83,86,87,89]	Not Considered	
12	CUSIP	Moderate Usage		Moderate Usage		Low Usage	
13	# GT	Only 1	[69,70,71,72,73,74,75,76,77,78,79,80,81,82]	Very high (10-4)	[83,84,85,86,87,88,89,90]	Average (1,2)	[80,91,92,93,94,95,96,97,98,99,100,101,102,103,104,105,106,107,108,109,110,111,112,113,114,115,116,117,118,119,120,121]
14	# Algorithm	🗶		🗸	[83,84,85,86,87,88,89,90]	🗶	
15	Type of Algorithm	🗶		-		🗶	
16	# Classifiers	Ranging from 1–4	[69,70,71,72,73,74,75,76,77,78,79,80,81,82]	Ranging from 1–9	[83,84,85,86,87,88,89,90]	Ranging from 1–10 [80,91,92,93,94,95,96,97,98,99,100,101,102,103,104,105,106,107,108,109,110,111,112,113,114,115,116,117,118,119,120,121]	
**SN**	**Attributes**	**Multiclass**		**Multi-label**		**Ensemble**	
**-**	**-**	**Characteristics**		**Characteristics**		**Characteristics**	
17	Classifier Type	SVM, RF, CNN DT, k-NN Agatston classifier, Elastic Net, NN, NB, XGBoost SVM, ELM, OAO, OAA, DDAG, ECOC [69,70,71,72,73,74,75,76,77,78,79,80,81,82]		RF, SVM, DT, KNN, LDA, LR, XGBoost, AdaBoost, GBA, Basic RNN, GRU RNN CNN, AAM [83,84,85,86,87,88,89,90]		kNN, GaussNB, LDA, QDA, RF, MLP, CNN, LSTM, GRU, BiLSTM, BiGRU Bagging, XGBoost, Adaboost, DNN, NB, NN, RS, GAMs, Elastic Net, GBMs, DT, CART, MARS, Logistic, EB, SMO, Boosting, MLDS, AVEn, MVEn, WAVEn, HTSA [80,91,92,93,94,95,96,97,98,99,100,101,102,103,104,105,106,107,108,109,110,111,112,113,114,115,116,117,118,119,120,121]	
18	# Classes	🗸	[69,70,71,72,73,74,75,76,77,78,79,80,81,82]	🗶		🗶	
19	Hyperparameters Used	🗸	[79]	🗸	[83,84,90]	🗸	[92,98,99,100]
20	Protocol	K-10	[64,65,66,67,68,69,70,71,72,73,74,75,76,77,78,79,80,81,82]	K-10, K, K-5	[83,84,85,86,87,88,89,90]	K-10, k, K-5	[80,91,92,93,94,95,96,97,98,99,100,101,102,103,104,105,106,107,108,109,110,111,112,113,114,115,116,117,118,119,120,121]
21	# PE parameters	Ranging from 1–5	[69,70,71,72,73,74,75,76,77,78,79,80,81,82]	Ranging from 1–8	[83,84,85,86,87,88,89,90]	Ranging from 1–8	[80,91,92,93,94,95,96,97,98,99,100,101,102,103,104,105,106,107,108,109,110,111,112,113,114,115,116,117,118,119,120,121]
22	Precision	🗸	[72,73,77,81,82]	🗶		🗸	[80,91,92,93,94,95,96,97,98,99,100,101,102,103,104,105,106,107,108,109,110,111,112,113,114,115,116,117,118,119,120,121]
23	PPV	🗶		🗸	[84,86]	🗸	[80,91,92,93,94,95,96,97,98,99,100,101,102,103,104,105,106,107,108,109,110,111,112,113,114,115,116,117,118,119,120,121]
24	NPV	🗶		🗸	[84,86]	🗸	[80,91,92,93,94,95,96,97,98,99,100,101,102,103,104,105,106,107,108,109,110,111,112,113,114,115,116,117,118,119,120,121]
25	FPR	🗶		🗸	[84,90]	🗸	[80,91,92,93,94,95,96,97,98,99,100,101,102,103,104,105,106,107,108,109,110,111,112,113,114,115,116,117,118,119,120,121]
26	FNR	🗶		🗸	[84]	🗸	[80,91,92,93,94,95,96,97,98,99,100,101,102,103,104,105,106,107,108,109,110,111,112,113,114,115,116,117,118,119,120,121]
27	Hamming Loss	🗶		🗸	[87]	🗶	
28	C-index	🗶		🗸	[83]	🗶	
29	Statistical Analysis	🗶		🗸	[83,84,85,86,87,88,89,90]	🗸	[80,91,92,93,94,95,96,97,98,99,100,101,102,103,104,105,106,107,108,109,110,111,112,113,114,115,116,117,118,119,120,121]
30	Power Analysis	🗶		🗸	[83,84]	🗶	
31	Hazard Analysis	🗶		🗸	[83]	🗶	
32	Survival Test	🗶		🗸	[83]	🗶	

SN: Serial number; SVM: Support vector machine; RF: Random forest; CNN: Convolutional neural network; DT: Decision tree, k-NN: k-Nearest neighbor; NN: Neural network; ELM: Extreme learning machine; OAO: One against one; OAA: One against all; DDAG: Decision direct acyclic graph; EOECC: Exhaustive output error correction code; LDA: Linear discriminant analysis; RNN: Recurrent neural networks; GRU: Gated recurrent unit; AAM: Algorithm adaptation methods; MARS: Multivariate adaptive regression splines; GAMs: Generalized additive models; PLR: Penalized logistic regression; GBM: Gradient boosted machines; MLP: Multilayer perceptron; CART: Classification and regression trees; SMO: Sequential minimal optimization; DNN: Deep neural network; NB: Naive Bayes; LSTM: Long short term memory network; EB: Ensemble boosting; MLDS: Multi-layer defense system; PPV: Positive predictive value; NPV: Negative predictive value; FPR: False positive rate; FNR: False negative rate; #GT: Number of ground truth.

## Appendix E. Performance Evaluation Metrics

### Performance Evaluation Metrics Descriptions

The PE for the multiclass and ensemble basically have accuracy (ACC), sensitivity (Sen), specificity (Spec), AUC, F1-Score which were calculated using values of true positives (TPs), false positives (FPs), false negatives (FNs), and true negatives (TNs). The formulae can be referred from Table A2. The performance evaluation for multi-label-based CVD is different as compared to multiclass and ensemble. They are label-based, instance-based performance evaluations.

In the label-based techniques, the PE parameters are checked for each label by the values of TPs, FPs, FNs, and TNs. All the labels have their own values. S, these are calculated by averaging methods (i) macro-averaging and (ii) micro-averaging [181]. The performance metrics say *β* is calculated by the values of TPs, FPs, FNs, and TNs, the macro-averaging techniques, macro-averaging (*β_macro_*) for all labels (*L*) is given by averaging *β* for each label “*p*”, as shown in Equation (A1).

(A1) $$\beta_{macro}=\frac{1}{L}\left(\overset{L}{\underset{p=1}{\sum }}\beta \;\left({\mathrm{TP}}_{p},{\;\mathrm{FP}}_{p},{\;\mathrm{TN}}_{p},{\;\mathrm{FN}}_{p}\right)\right)$$

In the same manner, for the micro-averaging techniques, the PE metrics are computed for each individual label and finally obtaining the micro-average (*β_micro_*) by using the Equation (A2).

(A2) $$\beta_{micro}=\beta \left(\overset{L}{\underset{p=1}{\sum }}{\mathrm{TP}}_{p},\overset{L}{\underset{p=1}{\sum }}{\mathrm{FP}}_{p},\overset{L}{\underset{p=1}{\sum }}{\mathrm{TN}}_{p},\overset{L}{\underset{p=1}{\sum }}{\mathrm{FN}}_{p}\right)$$

For instance-based performance evaluation, the parameters are calculated for individual instances, then the average value is computed and final the performance metric is performed. The final metric has a hamming loss, precision, recall, F1-score, Jaccard similarity coefficient score, and accuracy.

The multi-label dataset is supposed to be |E| with multi-label examples (pi, Qi), i = 1…|E|, and Qi ⊆ L, L is a set of all multiple labels. C is a multi-label classifier and Mi = C (pi) be the set of labels predicted by C. |E| indicates the features of the set E, while |Qi∩ Mi| indicates the feature of the intersection of true labels and the predicted labels. |Mi| indicates the features of predicted labels, and |Qi| indicates the features of the true labels.

Hamming loss shows the number of times when the label pair is misclassified. The lower value of Humming loss presents the better performance of the multi-label classifier. Jaccard score presents the ratio of the size of the intersection between predicted and the ground truth labels. Precision is the proportion of correct predictions out of all predictions. Likewise, recall is the ratio of correct predicted labels to the actual labels. F1-score is the combination of precision and recall Table A3.

**Table A2:** Performance evaluation metrics used in CVD risk assessment.

SN	Label-Based Performance Metrics	Mathematical Expression
1	Sensitivity (Sen), %	$\mathrm{Sen}=\left[\frac{\mathrm{TP}}{\left(\mathrm{TP}+\mathrm{FN}\right)}\right]\times 100$
2	Specificity (Spec), %	$\mathrm{Spec}=\left[\frac{\mathrm{TN}}{\left(\mathrm{TP}+\mathrm{FN}\right)}\right]\times 100$
3	Positive Predictive Rate (PPR), %	$\mathrm{PPR}=\left[\frac{\mathrm{TP}}{\left(\mathrm{TP}+\mathrm{FP}\right)}\right]\times 100$
4	Negative Predictive Rate (NPR), %	$\mathrm{NPR}=\left[\frac{\mathrm{TP}}{\left(\mathrm{TN}+\mathrm{FN}\right)}\right]\times 100$
5	False Predictive Value (FPV), %	$\mathrm{FPV}=\left[\frac{\mathrm{FP}}{\left(\mathrm{FP}+\mathrm{TN}\right)}\right]\times 100$
6	False Negative Value (FNV), %	$\mathrm{FNV}=\left[\frac{\mathrm{FN}}{\left(\mathrm{FN}+\mathrm{TP}\right)}\right]\times 100$
7	False Discovery Value, %	$\mathrm{FDV}=\left[\frac{\mathrm{TP}}{\left(\mathrm{TP}+\mathrm{FP}\right)}\right]\times 100$
8	F1-Score, %	$F1=\left[\frac{2\mathrm{TP}}{\left(2\mathrm{TP}+\mathrm{FP}+\mathrm{FN}\right)}\right]\times 100$
9	Accuracy (ACC), %	$\mathrm{ACC}=\left[\frac{\mathrm{TP}+\mathrm{TN}}{\left(\mathrm{TP}+\mathrm{FP}+\mathrm{TN}+\mathrm{FN}\right)}\right]\times 100$

**Table A3:** Performance evaluation metrics used in CVD risk assessment.

SN	Sample-Based Performance Metrics	Mathematical Expression
1	Hamming Loss, HL	$\mathrm{HL}\;\left(C,E\right)=\frac{1}{\left|E\right|}\overset{\left|E\right|}{\underset{i=1}{\sum }}\frac{\left|Q_{i}\Delta M_{i}\right|}{\left|L\right|}$
2	Jaccard Score, JS	$\mathrm{JS}=\frac{1}{\left|E\right|}\overset{\left|E\right|}{\underset{i=1}{\sum }}\frac{\left|Q_{i}\;\cap \;M_{i}\right|}{\left|Q_{i}\;\cup \;M_{i}\right|}$
3	Precision, Pe	$\mathrm{Pe}\;\left(C,\;E\right)=\frac{1}{\left|E\right|}\overset{\left|E\right|}{\underset{i=1}{\sum }}\frac{\left|Q_{i}\;\cap \;M_{i}\right|}{\left|\mathrm{Mi}\right|}$
4	Recall, Re	Re (C, E)=1|E|∑i=1|D||Qi ∩ Mi||Qi|
5	F1-score, F1	$F1\;\left(C,D\right)=\frac{1}{\left|E\right|}\overset{\left|E\right|}{\underset{i=1}{\sum }}\frac{2\left|Q_{i}\;\cap \;\mathrm{Mi}\right|}{\left|Q_{i}\right|+\left|\mathrm{Mi}\right|}$
6	$\mathrm{Subset}\;\mathrm{Accuracy},\;{\mathrm{Acc}}_{\mathrm{Subset}}$	${\mathrm{Acc}}_{\mathrm{Subset}}=\frac{1}{\left|E\right|}\overset{\left|E\right|}{\underset{i=1}{\sum }}I\left(M_{i}=Q_{i}\right)$

## Appendix F. Power Analysis

### Power Analysis for Multi-Label and Ensemble-Based CVD Risk Stratification

Power analysis can be done for multi-label and ensemble-based CVD systems. Its objective was to state the smallest data or sample size (*s*) needed to perform the multi-label, ensemble-based CVD risk classification. The parameters which are required for calculating power analysis are confidence interval, a margin error (*e*) as ±5%, and a sample proportion ($\hat{q}$), the *z*-score (*z*^∗^) (taken standard *z*-table). Therefore, the formula used is shown in Equation (A3) [264,265].

(A3) $$s={\left(\frac{z^{*}}{e}\right)}^{2}\times \hat{q}\;\left(1-\hat{q}\right)$$

## Appendix G. CVD Risk Assessment through Mobile, E-Health, and Cloud Techniques

### Characteristic of Mobile and Cloud-Based CVD Systems

**Table A4:** Characteristics of mobile and could-based CVD systems.

	**C0**	**C1**	**C2**	**C3**	**C4**	**C5**		**C6**	**C7**	**C8**		
**SN**	**Authors/Citations**	**ST**	**Year**	**Journal**	**DS**	**Diseases**		**FDA**	**SV**	**Comparator**		
1	Buss et al. [197]	SR	2020	JMIR	7 ED	CVD, DIA		🗶	🗶	No (i.e., standard care), await list control, intervention		
2	Villarreal et al. [198]	SR	2020	AIF	44	CVD		🗶	🗶	CVD, No CVD		
3	Xiao et al. [199]	R	2017	TM	151	CVD		🗶	🗶	CVD, No CVD		
4	Saba et al. [200]	R	2018	IHJ	100	CVD		🗶	🗸	CVD, No CVD		
5	Lillo-Castellano et al. [208]	R	2015	JBHI	6848	CVD		🗸	🗸	CVD, No CVD		
6	Huda et al. [201]	R	2020	TENSYMP	BIHAD	CVD		🗶	🗸	Normal ECG, Abnormal ECG		
7	Sakellarios et al. [209]	R	2018	EMBC	236	CAD		🗶	🗸	No CAD, OCAD, Non-OCAD		
8	Singh et al. [202]	R	2019	IEEEc	2	CVDa		🗶	🗸	Arrhythmia, CVD		
9	Spanakis et al. [203]	R	2020	EMBC	🗶	CHF		🗶	🗸	CHF, No CHF		
10	Paredes et al. [204]	R	2018	BIBM	1600	MI, CVD		🗶	🗸	Acute MI, No MI		
11	Freyer et al. [205]	R	2021	AJH	🗶	AF		🗶	🗸	AF, No AF		
12	Giansanti et al. [206]	S	2021	mHealth	🗶	CVD		🗶	🗶	Use of AI, non-use of AI		
13	Park et al. [207]	R	2014	IEEEa	🗶	Arrhythmia		🗶	🗶	Arrhythmia, CVD		
**SN**	**Authors/Citations**	**Non ML/ML**	**Cloud**	**Mob**	**Sea**	**DE**	**Analysis**	**# O**	**OT**	**# C**	**Classifier**	
1	Buss et al. [197]	Non-ML	🗶	🗸	🗸	🗸	🗸	2	Dia, CVD	3	🗶	
2	Villarreal et al. [198]	Non-ML	🗸	🗸	🗸	🗸	🗸	1	CVD	2	🗶	
3	Xiao et al. [199]	Non-ML	🗶	🗸	🗸	🗸	🗸	1	CVD	2	🗶	
4	Saba et al. [200]	Non-ML	🗸	🗸	🗸	🗸	🗸	1	CVD	2	🗶	
5	Lillo-Castellano et al. [208]	ML	🗸	🗶	🗸	🗸	🗸	1	CVD	2	k-NN	
6	Huda et al. [201]	ML, DL	🗸	🗸	🗸	🗸	🗸	1	Arrhythmia	2	SVM, CNN	
7	Sakellarios et al. [209]	ML	🗸	🗶	🗸	🗸	🗸	1	CVD	3	SVM	
8	Singh et al. [202]	DL	🗸	🗸	🗸	🗸	🗸	1	CVDa	2	CNN	
9	Spanakis et al. [203]	IoT	🗸	🗸	🗸	🗸	🗸	1	CHF	2	🗶	
10	Paredes et al. [204]	CI	🗶	🗸	🗸	🗸	🗸	2	CVD, MI	2	Bayesian	
11	Freyer et al. [205]	Non-ML	🗸	🗸	🗸	🗸	🗸	1	AF	2	🗶	
12	Giansanti et al. [206]	AI	🗸	🗸	🗸	🗸	🗸	1	CVD	2	🗶	
13	Park et al. [207]	ML	🗶	🗸	🗸	🗸	🗸	1	Arrhythmia	2	DT, RF	
	**C0**	**C19**	**C20**	**C21**	**C22**	**C23**	**C24**	**C25**	**C26**	**C27**	**C28**	**C29**
**SN**	**Authors/Citations**	**CV**	**Protocol**	**# PE**	**SEN**	**SPEC**	**Acc**	**Pre**	**F1 S**	**PV**	**SS**	**ROC**
1	Buss et al. [197]	🗶	🗶	0	🗶	🗶	🗶	🗶	🗶	🗶	🗶	🗶
2	Villarreal et al. [198]	🗶	🗶	0	🗶	🗶	🗶	🗶	🗶	🗶	🗶	🗶
3	Xiao et al. [199]	🗶	🗶	1	🗶	🗶	🗶	🗶	🗶	🗶	2.87	🗶
4	Saba et al. [200]	🗸	🗶	1	🗶	🗶	🗶	🗶	🗶	🗶	🗶	1
5	Lillo-Castellano et al. [208]	🗸	K	1	🗶	🗶	90	🗶	🗶	🗶	🗶	🗶
6	Huda et al. [201]	🗸	🗶	1	🗶	🗶	96	🗶	🗶	🗶	🗶	🗶
7	Sakellarios et al. [209]	🗸	🗶	3	44	98.7	85.1	🗶	🗶	🗶	🗶	🗶
8	Singh et al. [202]	🗶	🗶	1	🗶	🗶	97	🗶	🗶	🗶	🗶	🗶
9	Spanakis et al. [203]	🗶	🗶	1	🗶	🗶	1	🗶	🗶	🗶	🗶	🗶
10	Paredes et al. [204]	🗸	🗶	0	🗶	🗶	🗶	🗶	🗶	🗶	🗶	🗶
11	Freyer et al. [205]	🗸	🗶	1	🗶	🗶	1	🗶	🗶	🗶	🗶	🗶
12	Giansanti et al. [206]	🗸	🗶	0	🗶	🗶	🗶	🗶	🗶	🗶	🗶	🗶
13	Park et al. [207]	🗸	🗶	3	1	1	1	🗶	🗶	🗶	🗶	🗶

SN: Serial number; CV: Cross validation; SEN: Sensitivity; SPEC: Specificity; Acc: Accuracy; Pre: Precision; F1 S: F1 Score; PV: *p*-value; SS: Silberg score. DE: Data extraction; OT: Outcome types; C: Comparators; O: Outcomes; CI: Computational intelligence; CHF: Congestive heart failure; CVDa: CVD Auscultation; Dia: Diabetes; MI: Myocardial infarction; Mob: Mobile; Sea: Scientific validation; # O: Number of outcomes; # C: Number of classes. DS: Data size; BIHAD: MIT-BIH Arrhythmia Database; IEEEc: IEEE connect; AF: Atrial fibrillation; R: Research; SR: Systemic review; ST: Study type; IHJ: Indian Heart Journal; AIF: AI Foundation; TM: Telemedicine; IEEEa: IEEE-ACAINA; SV: Scientific validation; OCAD: Obstructive CAD; NonOCAD: Non-obstructive CAD.

## Appendix H. Miscellaneous Figures

### Appendix H.1. Anatomical Link between the Carotid Artery and Aortic Arch and Typical Neural Network

**Table A5:** Acronym.

SN	Abb *	Definition	SN	Abb *	Definition
1	ACC	American college of cardiology	42	IPN	Intraplaque neovascularization
2	AD	Alzheimer’s	43	KNN	K-nearest neighbor
3	AHA	American heart association	44	LBBM	Laboratory-based biomarker
4	AI	Artificial intelligence	45	LP	Label Powerset
5	ANOVA	Analysis of variance	46	LSTM	Long short-term memory network
6	APG	Acceleration Plethysmogram	47	LVD	Large vessel disease
7	ASCVD	Atherosclerotic cardiovascular disease	48	MCI	Mild cognitive impairment
8	AUC	Area-under-the-curve	49	MedUSE	Medication use
9	BCVD	Binary CVD	50	MI	Myocardial Infarction
10	BMI	Body mass index	51	ML	Machine learning
11	BR	Binary recursive	52	MLARM	Multi-label adaptive resonance asso ^&^ map
12	CAC	Coronary artery calcification	53	MLkNN	Multi-label k nearest neighbor
13	RetiCAC	Deep learning Retinal CAC score	54	MPH	Maximum plaque height
14	CAD	Coronary artery disease	55	MRI	Magnetic resonance imaging
15	CAS	Coronary artery syndrome	56	NPV	Negative predictive value
16	CC	Classifier chain	57	Non-ML	Non-machine learning
17	CCVRC	Conventional cardiovascular risk cal ^#^	58	OBBM	Office-based biomarker
18	CHD	Coronary Heart Disease	59	PCA	principal component analysis
19	CHD	Chronic Heart Conditions	60	PCE	Pooled cohort equation
20	cIMT	Carotid intima-media thickness	61	PE	Performance evaluation matrices
21	CKD	Chronic kidney disease	62	PMCI	Progressive MCI
22	CT	Computed tomography	63	PPV	Positive predictive value
23	CUSIP	Carotid ultrasound image phenotype	64	PTC	Plaque tissue characterization
24	CV	Cross-validation	65	QRISK3	QResearch cardiovascular risk algorithm
25	CVD	Cardiovascular disease	66	RA	Rheumatoid arthritis
26	CVE	Cardiovascular events	67	RakEL	Random k-label set
27	DL	Deep learning	68	#RC	Risk classes
28	DM	Diabetes mellitus	69	RF	Random forest
29	DT	Decision tree	70	RoB	Risk-of-bias
30	ECG	Electrocardiogram	71	ROC	Receiver operating-characteristics
31	EEGS	Event-equivalent gold standard	72	RRS	Reynolds risk score
32	ESC	European society of cardiology	73	SCD	Sudden cardiac death
33	FH	Family history	74	SCG	Seismocardiography (SCG-Z)
34	FNR	False-negative rate	75	SCORE	Systematic coronary risk evaluation
35	FPR	False-positive rate	76	SCMI	Significant memory concern
36	FRS	Framingham risk score	77	SMOTE	Synthetic minority over-sampling tech.
37	GCG	Gyrocardiography	78	SVM	Support vector machine
38	GUI	Graphical user interface	79	TPA	Total plaque area
39	HTN	Hypertension	80	US	Ultrasound
40	IM	Image modalities	81	WHO	World health organization
41	IMTV	Intima-media thickness variability	-	-	-

SN: Serial Number; Abb *: Abbreviation; ^#^ Calculator; ^&^ Asso. Associative; Tech.: Technique.
